# Species diversity and food web structure jointly shape natural biological control in agricultural landscapes

**DOI:** 10.1038/s42003-021-02509-z

**Published:** 2021-08-18

**Authors:** Fan Yang, Bing Liu, Yulin Zhu, Kris A. G. Wyckhuys, Wopke van der Werf, Yanhui Lu

**Affiliations:** 1 State Key Laboratory for Biology of Plant Diseases and Insect Pests, Institute of Plant Protection, Chinese Academy of Agricultural Sciences, Beijing, China; 2 University of Queensland, Brisbane, Queensland, Australia; 3 Centre for Crop Systems Analysis, Wageningen University and Research, Wageningen, The Netherlands

**Keywords:** Ecological networks, Ecosystem services, Agroecology

## Abstract

Land-use change and agricultural intensification concurrently impact natural enemy (e.g., parasitoid) communities and their associated ecosystem services (ESs), i.e., biological pest control. However, the extent to which (on-farm) parasitoid diversity and food webs mediate landscape-level influences on biological control remains poorly understood. Here, drawing upon a 3-year study of quantitative parasitoid-hyperparasitoid trophic networks from 25 different agro-landscapes, we assess the cascading effects of landscape composition, species diversity and trophic network structure on ecosystem functionality (i.e., parasitism, hyperparasitism). Path analysis further reveals cascaded effects leading to biological control of a resident crop pest, i.e., *Aphis gossypii*. Functionality is dictated by (hyper)parasitoid diversity, with its effects modulated by food web generality and vulnerability. Non-crop habitat cover directly benefits biological control, whereas secondary crop cover indirectly lowers hyperparasitism. Our work underscores a need to simultaneously account for on-farm biodiversity and trophic interactions when investigating ESs within dynamic agro-landscapes.

## Abstract (web summary)

Yang, Liu, Zhu et al. analyzed a dataset of 3-year parasitoid-hyperparasitoid trophic networks from 25 different agro-landscapes. Using path analysis, they uncover how species diversity and food web structure shape ecosystem functionality.

## Introduction

Biodiversity secures the sound functioning and stability of the world’s ecosystems^1–4^, though it is presently being lost at unprecedented rates due to land-use change, chemical pollution, and agricultural intensification^5,6^. As a central pivot within the interplay between agri-food production and ecosystem service (ES) delivery^7^, insect biodiversity underpins globally important services such as pollination and biological pest control^8,9^, which are valued at US $14 and $24 ha^−1^ y^−1^, respectively^10^. Alleviating the root causes of insect biodiversity loss carries broad societal benefits, as it can help restore ES delivery, improve resource-use efficiencies, raise the economic solvency of farming operations, and bolster ecological resilience in the face of global change^11,12^.

The conversion of natural habitats to simplified, genetically uniform crop fields is a well-recognized driver of insect biodiversity loss. While more diverse landscape mosaics buffer against species loss for certain taxa^13,14^, ES-providing organisms, such as insectivorous predators and parasitoids, do not exhibit consistent responses to landscape composition^15^. Within individual cropping fields, biological control is affected by various aspects of agricultural intensification^16^, i.e., the incorporation of plant diversity^8,17^, agronomic management such as tillage^18^ or agro-chemical use^19^. Landscape composition equally shapes ecosystem disservices (EDSs) such as pest colonization^20^, hyperparasitism^21^, and intraguild predation^22^ or those provided by entomopathogenic fungi^23^—all of which interfere with on-farm biological control^24^. For example, EDS providers such as hyperparasitoids thrive in complex landscapes^25^, and their action can destabilize parasitoid communities and dampen overall parasitism^26^. Overall, the net effects of landscape complexity are highly variable^27^, and the resulting impacts on ES or EDS delivery are unclear^28^, thus complicating efforts to reliably forecast biological control or pest infestation pressure. However, this absence of consistent relationships between landscape make-up and ecosystem functionality can be resolved by adopting a multitrophic food web perspective^29^.

Food webs describe species interactions within and between various trophic levels, and their composition dictates biodiversity-ecosystem functionality^30,31^. As a key food web metric, network generality (i.e., mean number of host or prey species per consumer) mediates ESs^32^, with high generality entailing the presence of multiple prey or host items for each consumer (i.e., predator or parasitoid) within the food web^33,34^ and thereby mitigating impacts of eventual species loss^35^. Conversely, food web vulnerability (i.e., mean number of consumers per host or prey) indicates how multiple consumers share one single prey or host item, thus increasing competition for resources and eventually causing secondary extinction^36^. To date, host-parasitoid models have been widely used to characterize food web structure^37^ due to ease of sampling, quantitative interpretation of (multitrophic) interaction networks^38^, and advances in DNA-based molecular detection^39,40^. So far, this approach has allowed capturing the direct effect of landscape-level variables on food web structure and ESs such as parasitism^41^ as well as EDSs, i.e., hyperparasitism. However, the necessary insights regarding how particular food web features mediate landscape-level impacts on ES delivery are lacking.

In general, landscape complexity favors biodiversity and often enhances biological control^42^, while on-farm management affects myriad food web features^43,44^. However, there is only scant knowledge regarding the extent to which food web complexity affects the abundance of biological control organisms and EDS providers, particularly within the highly dynamic and disturbance-prone context of agro-ecosystems^45^. Although on-farm biological control relates to metrics such as community evenness, linkage strength, and network centrality^46^, these patterns do not necessarily hold across cropping systems and landscape contexts. By disentangling how species diversity and food web complexity jointly mediate landscape-level impacts on biological control, one could facilitate the formulation of universal theorems. Farming systems in northern China are managed intensively by smallholders, and the resulting agro-landscapes exhibit high levels of diversity and fragmentation^47^, i.e., diversified secondary crop cultivation. In local cotton crops, aphids (Hemiptera: Aphididae) are a focal pest, and hymenopteran parasitoids are key biological control agents^48^. On-farm management and landscape context determine aphid colonization rates and the action of EDS providers, e.g., hyperparasitoids^49^. The aphid-parasitoid network structure is equally influenced by landscape complexity and management practices such as pesticide or fertilizer applications^50^.

Here, drawing upon multiyear observational surveys in China’s cotton agro-landscapes, we examine how landscape composition affects (1) the species diversity of different ES and EDS providers, (2) food web structure and (3) the resulting ESs or EDSs, i.e., biological control or hyperparasitism. Furthermore, using path analysis with structural equation modeling (SEM), we reveal how on-farm food webs mediate landscape-level impacts on biological control. Our work shows how an in-depth characterization of food web structure helps clarify the determinants of ecosystem functionality (ESs and EDSs) and can ultimately guide the design and deployment of ecologically based pest management strategies at the landscape level.

## Results

### Aphid-parasitoid diversity and tri-trophic food web structure

**Fig. 1:**
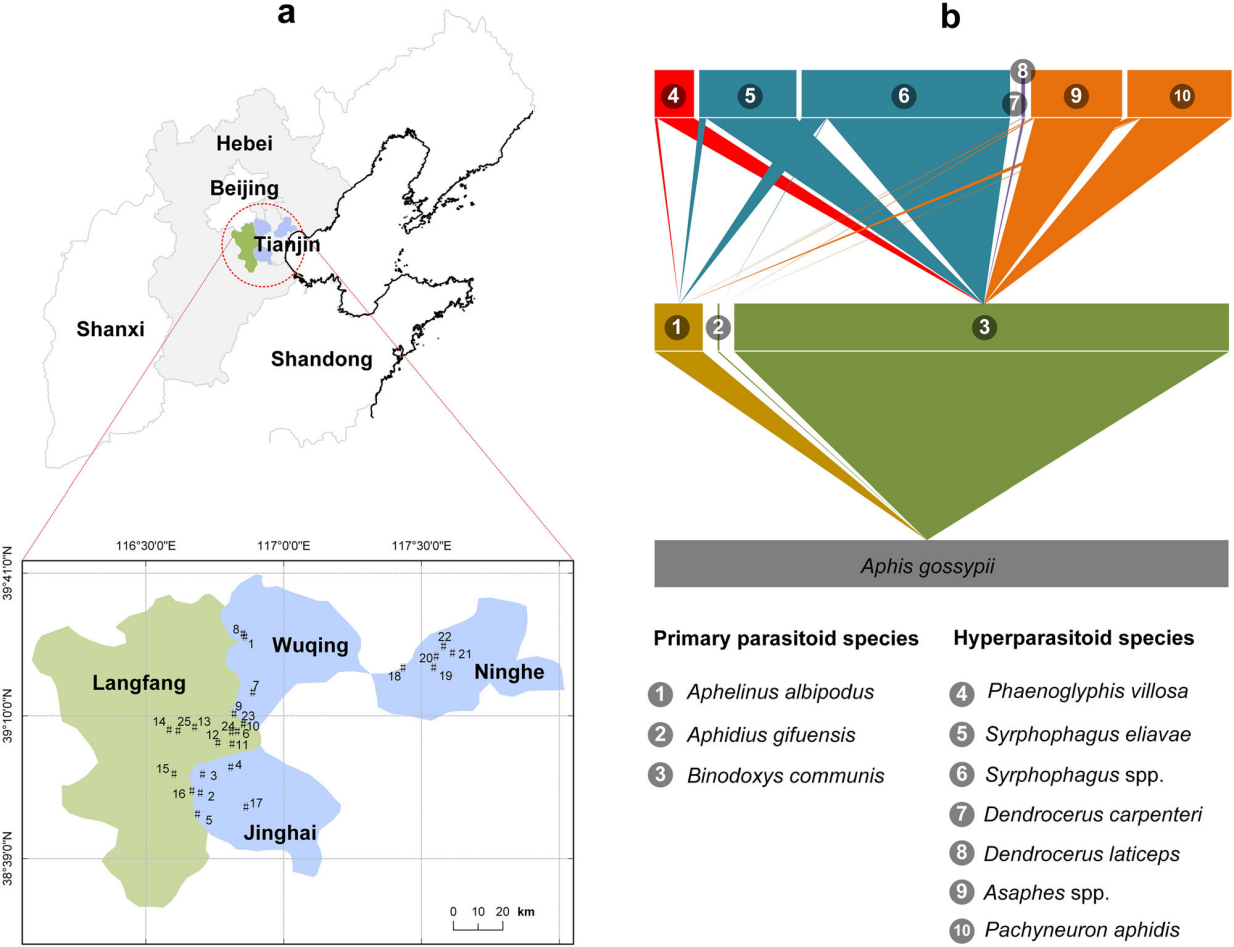
Geographic distribution of study sites in northern China and aphid-primary-hyperparasitoid tri-trophic food web. From 2014 to 2016, a total of 25 sites were identified across four geographic regions in northern China (**a**). **b** Diagrams the overall quantitative food webs including three trophic levels, with the lowest level (gray bar) comprising herbivorous hosts, i.e., the cotton aphid *Aphis gossypii*. Species numbered 1–3 are the primary parasitoids (middle trophic level): *Aphelinus albipodus*, *Aphidius gifuensis* and *Binodoxys communis*; and 4–10 are the hyperparasitoid species (upper trophic level): *Phaenoglyphis villosa*, *Syrphophagus eliavae*, *Syrphophagus* spp., *Dendrocerus carpenteri*, *Dendrocerus laticeps*, *Asaphes* spp., *Pachyneuron aphidis*. Species that are marked with the same color belong to the same family. The width of a given triangle reflects the relative proportion of linkage effects.

Throughout the 3-year study, a total of 2153 mummified (i.e., parasitized) aphids were collected from 25 different sites in northern China (Fig. 1a). DNA-based species identification and food web assembly revealed how 2503 parasitoid and hyperparasitoid individuals (11 species) were involved in 2386 distinct trophic interaction events. These included one target aphid pest (*Aphis gossypii*), 3 species of primary parasitoids (1569 individuals), and 7 species of hyperparasitoids (934 individuals) (Fig. 1b). The primary parasitoid community consisted mainly of *Binodoxys communis* (Braconidae) (average ± SE as 91% ± 2% of individuals), while *Syrphophagus* spp. (Encyrtidae) constituted 40% ± 4% (average ± SE) of hyperparasitoid taxa. The aphid-parasitoid-hyperparasitoid food web was highly stable over the years (see Supplementary Fig. 1).

### Direct effects of food web features on ecosystem services (ESs) and disservices (EDSs)

We first assessed the direct effects of three key quantitative metrics of primary-hyperparasitoid food web generality (Gq), vulnerability (Vq), and connectance (Cq) on selected ESs (parasitism rate on *A. gossypii*) and EDSs (hyperparasitism rate) (Table 1). General linear model (GLM) analysis with multiple model selection inference showed that the best model (*Δ*AICc = 0) contained the unique predictor generality, which was negatively related to ESs (i.e., parasitism rate) (*P* = 0.020, Supplementary Table 2), whereas food web vulnerability was positively related to EDSs (i.e., hyperparasitism rate) (*P* = 0.007, Supplementary Table 2).

**Table 1:** Summarized effects of predictors on ecosystem services and food web metrics.

Method	Predictor	Response variable	Coeff^b^	*P*
Linear regression	EDS	ES	0.10	0.158
GLM	Gq	ES	−0.13	**0.020**
GLM	Vq	EDS	0.12	**0.007**
GLM	NCH	ES	0.25	0.066
GLM	Maize	EDS	0.44	0.061
	NCH	EDS	0.88	0.077
GLM	Intercept^a^	Gq	1.26	0.561
GLM	SC	Vq	−3.21	**0.034**
GLM	Intercept	Parasitoid richness	1.84	**<0.001**
GLM	Cotton	Parasitoid diversity	−0.80	0.113
	SC	Parasitoid diversity	0.68	0.062
GLM	Intercept	Parasitoid richness	4.12	**<0.001**
GLM	Intercept	Hyperparasitoid diversity	0.11	**<0.001**
LMM	Parasitoid richness	ES	−0.06	**0.001**
	NCH	ES	0.20	0.061
LMM	Vq	EDS	0.11	**0.010**
	NCH	EDS	0.57	0.076
Path analysis	Parasitoid richness	ES	−0.524	**0.043**
	Parasitoid diversity	ES	0.072	0.859
	Gq	ES	−0.163	0.705
	Gq	Parasitoid richness	0.693	**0.000**
	Parasitoid diversity	Gq	0.899	**0.000**
Path analysis	Vq	EDS	0.514	**0.009**
	Hyperparasitoid diversity	Vq	0.824	**0.000**
	SC	Vq	−0.449	**0.000**
	Hyperparasitoid richness	Hyperparasitoid diversity	0.778	**0.000**

Analyses include linear regression, generalized linear model (GLM), linear mixed effect model (LMM), and path analysis with structural equation models (SEM). Effects are assessed of multiple predictors on either ecosystem services (ES; parasitism) or disservices (EDS; hyperparasitism). Detailed descriptions of all variables are provided in Supplementary Table 1.

Gq and Vq are the generality and vulnerability of primary-hyperparasitoid food web; NCH is non-crop habitat cover, SC is secondary crop cover.

^a^Best model without landscape variables.

^b^Regression effect coefficient.

### Direct effects of landscape composition on different response variables

We tested the direct landscape effects on ESs (parasitism rate) and EDSs (hyperparasitism rate), food web features, and parasitoid diversity individually (Table 1). Landscape factors were previously selected based on correlation analysis and principal component analysis (PCA) (Supplementary Fig. 2). GLM analyses and model selection inference showed that landscape factors had no direct influences on ESs and EDSs, although the percentage of non-crop habitat (NCH) cover was (marginally) negatively related to ESs (conditional average: *P* = 0.066) but positively related to EDSs (conditional average: *P* = 0.077, Supplementary Table 3). Additionally, no landscape factors were directly related to food web generality (*P* = 0.561), although secondary crop cover (SC) was negatively related to food web vulnerability (*P* = 0.034, Supplementary Table 4). No landscape factors were related to the species richness or community diversity (Shannon diversity) of either primary parasitoids or hyperparasitoids (Supplementary Tables 5 and 6).

### Direct effects of combinational predictors on ecosystem functionality

Earlier GLM analyses allowed for an initial identification of the direct effects of several predictors on ESs and EDSs. However, as ecosystem functionality is determined by a multitude of factors, linear mixed effect model (LMM) analysis helped assess the direct effects of combinational predictors belonging to three groups: landscape composition, species richness and diversity, and food web features, on ESs and EDSs (Table 1; Supplementary Tables 7–10). The parasitism rate was negatively related to species richness (conditional average: *P* = 0.002, Supplementary Table 8) but not to the Shannon diversity of primary parasitoids (*P* = 0.864). Food web generality was not related to ESs (*P* = 0.685) when simultaneously considering other effects of predictors. Additionally, landscape variables such as the percentage of SC (*P* = 0.242) and cotton area cover (*P* = 0.736) had no direct effects on ESs. In particular, NCH cover remained (marginally) positively related to ESs (*P* = 0.055, Supplementary Table 8). For hyperparasitism, food web vulnerability was positively related to EDSs (conditional average: *P* = 0.040, Supplementary Table 10). NCH had a (marginally) positive effect on EDSs (*P* = 0.087), while no effects were found for other predictors, such as hyperparasitoid species richness (*P* = 0.103) and community diversity (*P* = 0.199).

### Path analysis for assessing cascading effects

As a first step, the above GLMs and LMMs permitted the identification of the direct effects of several predictors on the respective ES or EDS of aphid parasitism or hyperparasitism. Next, a path analysis with structural equation modeling (SEM) quantified their combined effects and the eventual cascaded relationships between various factors and ecosystem functionality (i.e., ESs or EDSs). Direct linear regression analysis showed that the parasitism rate was not directly related to the hyperparasitism rate (Table 1; Supplementary Fig. 3), and we thus assessed both ESs and EDSs individually.

**Fig. 2:**
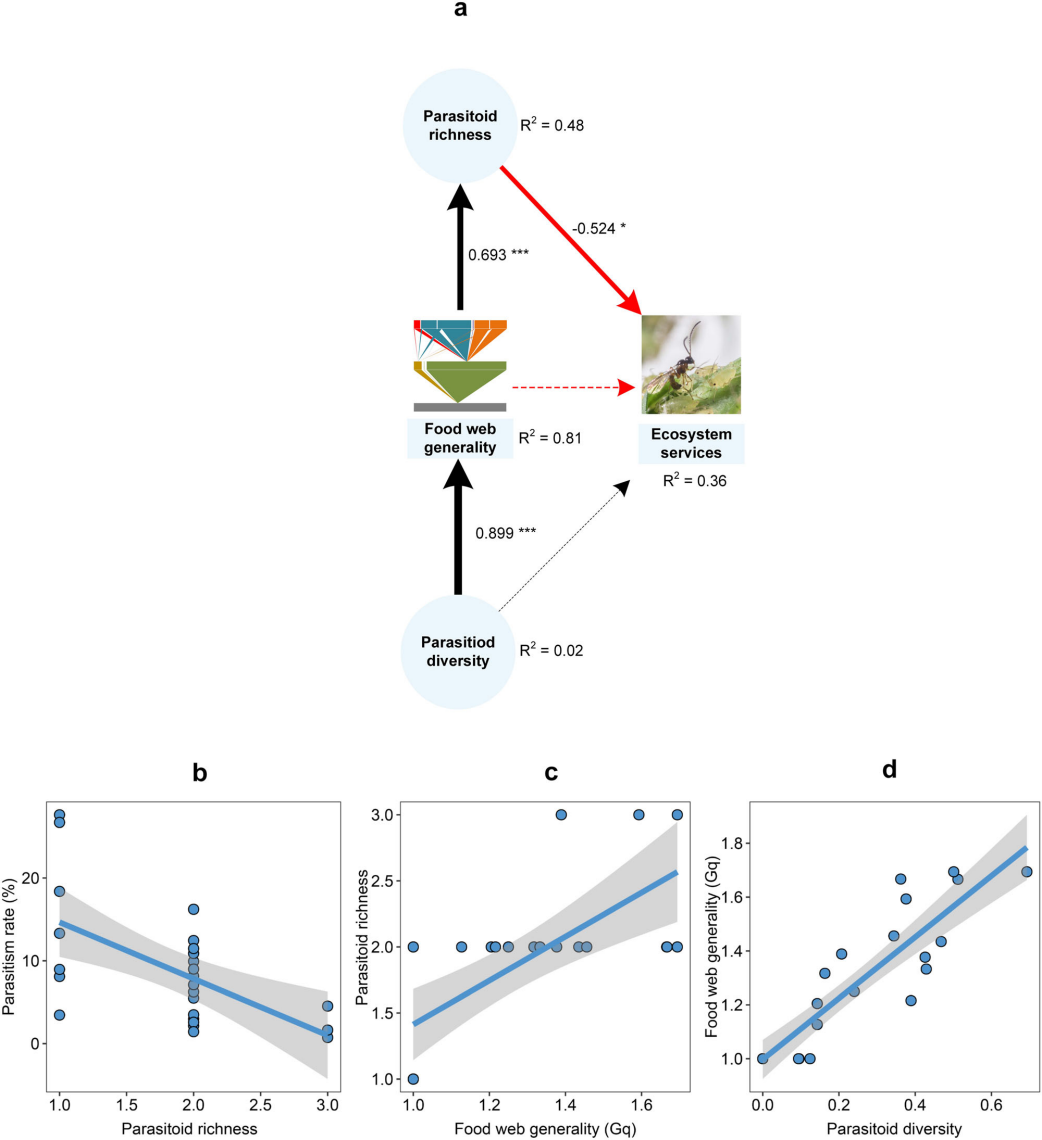
Causal paths between the ecosystem service (ES) of biological control and different predictors. In the SEM analysis, ES is the ultimate response variable, while parasitoid richness, parasitoid diversity and food web generality (Gq) are both predictors and response variables. The paths reveal both direct and indirect relationships between individual predictors and response variables. **a** Shows the paths after removing non-crop habitat (NCH). Standardized coefficients are shown for each path and scaled as line width. Black and red lines indicate either positive or negative relationships, with solid lines representing statistically significant effects and dotted lines showing nonsignificant effects (*P < 0.05; **P < 0.01; ***P < 0.001). *R*^2^ shows the explanatory proportion of the total variance for each response variable in the model (Supplementary Table 11). **b**–**d** Show significant relationships based on SEM analysis, with solid lines and shaded zones indicative of the regression lines and 95% confidence intervals (*n* = 25), respectively.

For parasitism (ES), we first entered all four predictors (NCH, species richness, Shannon diversity, food web generality) from the best model into the SEMs (Supplementary Fig. 5; Supplementary Table 11). The parasitism rate was (directly) negatively related to the species richness of primary parasitoids (*β* = −0.490, *P* = 0.043, Supplementary Fig. 5). However, the parasitism rate was not (directly) affected by food web and landscape features such as generality (*β* = −0.086, *P* = 0.708) and NCH (*β* = 0.295, *P* = 0.088), respectively. Additionally, NCH had no effect on the species richness (*β* = −0.055, *P* = 0.725) and diversity (*β* = −0.156, *P* = 0.455) of primary parasitoids or on the food web generality (*β* = 0.019, *P* = 0.843). However, a direct positive relationship was found between food web generality and species richness (Fig. 2c). Moreover, a high diversity of primary parasitoids predicted higher food web generality (*β* = 0.902, *P* < 0.001, Fig. 2d), thus indirectly predicting richer parasitoid species and lower parasitism. After removing NCH (which linked nonsignificant paths) and rerunning SEM analysis, the results were consistent with those from earlier analyses (Fig. 2a; Table 1; Supplementary Table 11). The total effect of species richness on the ES was −0.524, and those of generality and diversity were −0.363 and −0.326, respectively (Table 2 and Fig. 2a). Hence, parasitoid-mediated biological control was directly attenuated by parasitoid richness (Fig. 2b) but indirectly modulated by food web generality and parasitoid diversity.

**Table 2:** Summarized effects of different predictors on ultimate ecosystem functionality.

Ecosystem functionality	Predictor	Direct effect	Indirect effect	Total effect
ES (parasitism)	Parasitoid richness	−0.524		−0.524
	Food web generality (Gq)		−0.363	−0.363
	Parasitoid diversity		−0.326	−0.326
EDS (hyperparasitism)	Food web vulnerability (Vq)	0.514		0.514
	Hyperparasitoid diversity		0.424	0.424
	Hyperparasitoid richness		0.329	0.329
	Secondary crop cover (SC)		−0.231	−0.231

**Fig. 3:**
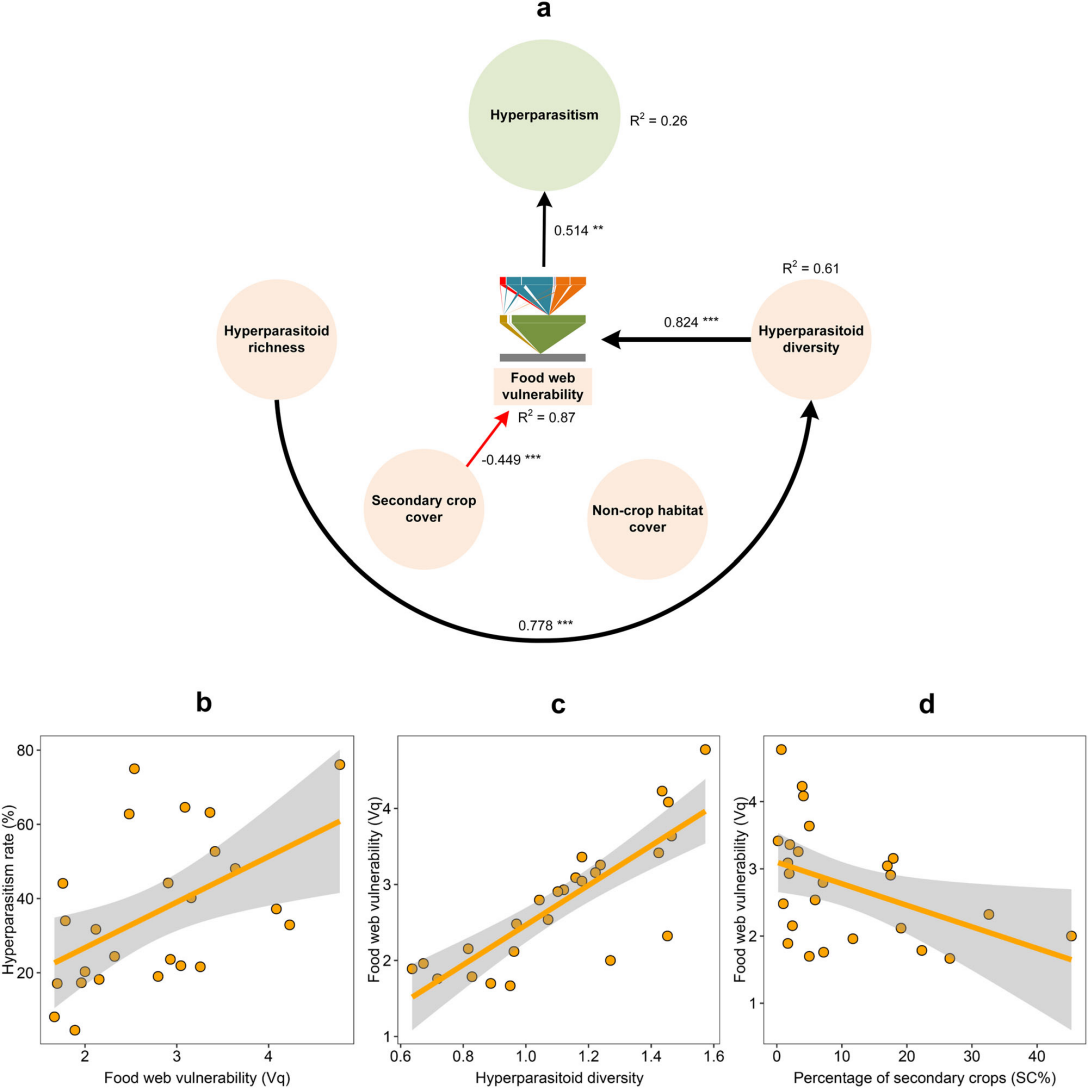
Causal paths between the ecosystem disservice (EDS) of hyperparasitism and different predictors. In the SEM analysis, EDS is the ultimate response variable, while hyperparasitoid richness and diversity and food web vulnerability (Vq) are both predictors and response variables. Non-crop habitat (NCH) and secondary crop cover (SC) are exogenous variables. The paths reveal both direct and indirect cascading relationships between predictors and response variables. **a** Shows the paths after removing all nonsignificant paths. Standardized coefficients are shown for each path and scaled as line width. Black and red lines indicate either positive or negative relationships, with solid lines representing significant effects and dotted lines showing nonsignificant effects (*P < 0.05; **P < 0.01; ***P < 0.001). *R*^2^ shows the explanatory proportion of the total variance for each response variable in the model (Supplementary Table 12). **b**–**d** Show the significant relationships based on SEM analysis, with solid lines and shaded zones indicative of regression lines and 95% confidence intervals (*n* = 25), respectively.

For the ecosystem service (ES, parasitism rate), results were based on the last SEM analysis after removing the landscape variable (Fig. 2a). Parasitoid richness had a direct effect on the studied ES, whereas food web generality (Gq) and parasitoid diversity had indirect effects (calculated as indirect path effect products). For the studied ecosystem disservice (EDS, hyperparasitism rate), food web vulnerability (Vq) had a direct positive effect on hyperparasitism, while hyperparasitoid richness and diversity had cascading positive effects (Fig. 3a). Secondary crop cover (SC) had a cascading negative effect on EDS. Total effects are computed by summing the direct and indirect effects for each predictor.

A similar path analysis was drawn for hyperparasitism (an EDS), in which we entered five predictors (NCH, secondary crops, hyperparasitoid richness and their Shannon diversity, and the food web vulnerability) from the best model into the SEMs (Supplementary Fig. 6; Supplementary Table 12). The hyperparasitism rate was (directly) positively related to food web vulnerability (*β* = 0.415, *P* = 0.044), whereas other predictors had no direct effects (Supplementary Fig. 6). Hyperparasitoid diversity and the landscape-level SC cover had respective (direct) positive or negative effects on food web vulnerability (*β* = 0.817, *P* < 0.001; *β* = −0.424, *P* < 0.001), respectively. NCH did not affect the other response variables. Hyperparasitoid richness was predictive of community diversity (*β* = 0.829, *P* < 0.001), thus indirectly enhancing hyperparasitism. After removing the nonsignificant paths from the SEMs, the results were consistent with those of earlier analyses (Fig. 3a, Table 1; Supplementary Table 12). Moreover, food web vulnerability had the highest indirect effect on several paths and directly influenced hyperparasitism (total effect 0.514; Table 2; Fig. 3a, b). Hyperparasitoid diversity also had a positive effect on food web vulnerability (Fig. 3c) and indirectly enhanced hyperparasitism (total effect 0.824*0.514 = 0.424; Fig. 3a; Table 2). The landscape-level SC cover exhibited a direct negative effect on food web vulnerability (Fig. 3d) and indirectly lowered hyperparasitism (total effect −0.449*0.514 = −0.231; Fig. 3a; Table 2). Hence, the EDS of hyperparasitism was shaped by food web vulnerability and modulated by agro-landscape composition and parasitoid diversity.

## Discussion

In global farming systems, food web complexity and insect-mediated ESs, such as biological control, are tied to on-farm plant diversity, management intensity, and the surrounding landscape matrix. As ES-providing organisms such as insect parasitoids exhibit inconsistent responses to landscape-level variables, often, biological control cannot be reliably predicted across landscape complexity gradients^15^. Here, we illustrated how particular features of insect food webs and diversity metrics modulated the effects of land-use variables on ecosystem functionality, i.e., aphid biological control. More specifically, path analysis revealed how parasitoid richness—as shaped by food web generality—and diversity mediated landscape-level determinants of biological control. However, non-crop habitat cover exerted no effects on parasitoid diversity and food web generality. Moreover, parasitism was attenuated by parasitoid richness and indirectly weakened by food web generality. A diverse parasitoid community could thereby dampen biological control. Hyperparasitism is an important EDS, with hyperparasitoids often compromising the role of primary parasitoids in biological control^51^. In our study, hyperparasitism was directly strongly tied to food web vulnerability, with the latter parameter directly affected by the landscape-level SC (secondary crops) cover and hyperparasitoid diversity. While SC interfered with hyperparasitism, its effects were counteracted by hyperparasitoid diversity.

Ecosystem functionality is usually shaped by multiple biotic and abiotic factors. In farming landscapes, non-crop habitat tends to lift the population levels of beneficial organisms and benefit biological control^52^. Non-crop habitat provides shelter, alternative host items, and carbohydrate-rich foods as delivered by pollen- or nectar-bearing plants^53^, which are resources that are often scarce in ephemeral, disturbance-prone agro-ecosystems^20^. As such, non-crop habitat can benefit aphid parasitoid populations^54^ and bolster parasitism levels^55^, which in turn lower pest damage and increase crop yields^9^. However, landscape-level crop heterogeneity tends to benefit parasitoid richness and can increase the level of biological control^56^. Natural enemies exhibit taxa-specific responses to landscape context and ecosystem alterations^55,57^. For instance, primary parasitoids thrive in complex landscapes, whereas simple landscapes often support diverse hyperparasitoid communities^45^. However, in our study, landscapes with more non-crop habitat exhibited only marginal increases in parasitism levels. Conversely, diverse farming landscapes (i.e., high secondary crop cover) were typified by lower food web vulnerability and attenuated hyperparasitism. Hence, crops such as peanut, soybean, sweet potato, vegetables, or fruit trees likely provide a suite of (food, non-food) resources that disproportionately favor primary parasitoids. This characteristic underscores an urgent need to clarify the relative contribution of non-crop habitat compared to that of other field- and landscape-level parameters. Ideally, these multitrophic food web ecology studies are conducted in cereal systems, where aphids and their parasitoid species are exceptionally well studied^45,52,54^ and are more speciose than are temperate cotton systems^40^.

In both natural and anthropogenic ecosystems, land-use change can directly affect the host-parasitoid food web structure^43,58^. In our study, however, no (direct, indirect) effects of landscape composition on food web generality were detected. However, food web generality modulated the effects of parasitoid diversity on the parasitism rate (Fig. 2). Complex food webs (i.e., high generality) exhibited more links between trophic levels (Supplementary Fig. 4a) and, in settings with diverse (primary) parasitoid communities, thus reduced parasitism rates. However, during aphid outbreaks, the latter metric can be skewed by exponentially increasing aphid numbers (denominator) compared to parasitoid mummies (numerator)^54^. Additionally, in settings with complex food webs, primary parasitoids sustain more hyperparasitoids and potentially reduce their interspecific competition in the food web due to down-top effects (prey affect natural enemies) (Supplementary Fig. 4a). A second food web metric, i.e., food web vulnerability also exerted important impacts on hyperparasitism. In settings with high food web vulnerability, hyperparasitoid richness was related to primary parasitoid diversity and ultimately enhanced the hyperparasitism rate (Fig. 3), which may have been caused by resource shortages, thus potentially increasing interspecific competition due to top-down effects (natural enemies attack prey) in the food web (Supplementary Fig. 4b).

Food webs represent networks of different trophic relationships, with a weighted generality metric related to the food web universality of the lower trophic level, the diversity of host items for the upper trophic level (i.e., hyperparasitoids) and the overall robustness of species interactions^59^. Food web vulnerability predicts trophic fragility^33^, in which more than one species in the upper trophic level shares one common resource item. Once the food web structure is well characterized, its implications can be assessed in terms of ecosystem functionality^60^. In our study, food web generality was positively related to parasitoid richness and negatively impacted their associated ESs (i.e., in-field parasitism rate), irrespective of landscape context. Our findings thus conflicted with existing research results^45,49^ in which landscape complexity has been deemed to be a key determinant of aphid-parasitoid food web structure^61^. More complex food webs with diverse interactions can foster stability^62^ through trophic complementarity^63^, although individual species could lend stability^64^ and facilitate species coexistence^65^. In our study, complex food webs sustained species-rich hyperparasitoid communities and thereby restrained biological control and destabilized ecosystem functioning^34^. Although food web vulnerability directly affected the hyperparasitism rate, no linear relationship was detected between parasitism and hyperparasitism (Supplementary Fig. 3). Therefore, the primary and hyperparasitoid species that comprise the trophic interactions of on-farm food webs are expected to differentially contribute to ESs (i.e., biological control) or EDSs (i.e., hyperparasitism).

By characterizing the extent to which food web structure mediates landscape-level impacts on ES delivery, we found that network generality played a pivotal role in determining aphid biological control. Conversely, EDSs (i.e., hyperparasitism) were dictated by network vulnerability and further modulated by landscape features (i.e., secondary crop cover) and (hyper)parasitoid diversity. Our assessment is, however, constrained by a number of elements, e.g., the species-poor herbivore community in China’s cotton agro-ecosystems and a rather simplified quantification of parasitoid-mediated pest suppression. Nevertheless, our multitrophic food web analytical approach constitutes a powerful lens to quantitatively assess the relative contributions of different (on-farm, landscape-level) determinants of ES delivery. Our findings showed that the active conservation of non-crop habitat (e.g., natural habitats, hedgerows, flower strips) or landscape-level crop heterogeneity could bolster parasitism rates and simultaneously enhance the pest control action of other organisms^66^. By accentuating the contribution of species diversity and food web structure, our work can help refine ecological intensification schemes, guide landscape-level interventions to restore natural biological control, or amend existing “area-wide” agri-environment schemes^67^. Our food web approach also enables a more complete accounting of farm management, e.g., insecticide use, impacts on ESs, and permits an in-depth assessment of how (smallholder) farmers either bolster or degrade ecosystem functionality. Aside from enabling a step-change in applied agro-ecological research, our empirically derived findings can help mitigate mounting anthropogenic pressures on agro-biodiversity and their associated ESs in China and internationally.

## Methods

### Study sites and landscape characterization

To assess the landscape-level effects on aphid parasitism and host-parasitoid food webs, we selected 25 different sites across a landscape gradient in the >3000 km^2^ cotton-growing region in China’s Hebei and Tianjin Provinces (116°30′−117°50′E, 38°39′–39°41′N). From 2014 to 2016, 7–10 sites were selected each year and spaced at a minimum distance of 3 km to avoid spatial autocorrelation (Fig. 1a). Per site, 1500-m radius landscape sectors from the focal cotton field were digitized. Google Earth and land-use categories were defined by ground truthing, and the position of each focal cotton field was recorded using a handheld GPS unit (Model MG768, Beijing UniStrong Science & Technology Co. Ltd., China). Imagery was digitized by using ArcGIS 10.2 (ESRI, Environmental Systems Research Institute, Inc., USA), and each landscape was classified into five land-cover types: (1) cotton, (2) maize, (3) secondary crops (i.e., soybean, peanut, sweet potato, vegetables, fruit orchards), (4) non-crop habitat (i.e., grassland, shrubs, forest), and (5) urban (i.e., roads, cemented hard surface including buildings, water and abandoned land). The proportion of each land cover type was quantified using Fragstats 4.0 software^68^. As a measure of landscape diversity, we used Simpson’s inverse diversity index (SIDI), calculated as SIDI = 1/Σ(_*pi*_)^2^, in which _*pi*_ is the proportion of each land-use category within each 1500-m radius of cotton agro-landscape^69^.

### Parasitism and hyperparasitism rate

Parasitoid-mediated biological control (i.e., an ES) was quantified using the parasitism rate, or more specifically, the proportion of mummified aphids among all aphids (i.e., mummies and live aphids). At each site, the numbers of aphids and parasitoid mummies were recorded on 50 cotton plants in each of three randomly selected plots (min. 1000 m^2^) within the focal cotton field, with each plot at a min. 10 m distance from the field border to avoid potential edge effects. Within each plot, five points were randomly chosen using a Z-shaped sampling grid, and 10 plants were inspected at each point. In each field, sampling was carried out three times (at 7–10-day intervals) from early July to mid-August when cotton aphids tended to reach outbreak levels^70^. In each plot, mummified aphids were collected over a 15-min sampling window and individualized within 1.5-mL centrifuge tubes with 95% ethanol. Next, samples were kept at −20 °C for future PCR-based parasitoid identification. Sampling was exclusively performed in insecticide-free cotton plots. The focal fields were managed without pesticides during the whole study period, and farmers were financially compensated for any yield loss that resulted from this modified management regime.

The hyperparasitism rate (i.e., an EDS) was calculated as the proportion of hyperparasitoids detected from mummy samples. If PCR-based parasitoid identification revealed the presence of one or more hyperparasitoids from one given mummified sample, the respective hyperparasitism rate was defined as 1. In the absence of hyperparasitoid DNA for a mummified sample, the hyperparasitism rate was maintained at 0.

### Food web construction and parasitoid diversity

The DNA of mummified aphids was extracted using a modified Chelex extraction method. Next, multiplex and single PCRs were jointly used to detect aphid and parasitoid species, as in Zhu et al.^40^. This method can detect the DNA of both parasitoid and hyperparasitoid species in parasitized (or mummified) aphids^39^. Data were used to determine the abundance, richness, and community diversity (Shannon’s diversity, H′) of primary parasitoid and hyperparasitoid species. Abundance reflected the total number of parasitoids at different trophic levels. Richness was the total number of species, while Shannon’s diversity was calculated as^71^ by using the “picante” package^72^ in R 4.0.2 software^73^:

1 $${{{{{\rm{H}}}}}}^{\prime} =-\mathop{\sum }\limits_{i=1}^{S}{p}_{i}{ln}{p}_{i}$$

in which *S* is the species number and _*pi*_ is the proportion of species *i*.

For each study site, we assembled quantitative food webs. As the cotton aphid *A. gossypii* was the only host for resident parasitoids at our study sites, the ecological network assembly was focused on the primary parasitoid-hyperparasitoid food webs. The structure of each food web was characterized using three quantitative metrics: weighted generality (G_q_), vulnerability (V_q_), and connectance (C_q_). The above metrics are commonly used to describe the interaction and complexity in ecological networks, including host-parasitoid food webs^43^. The weighted quantitative metrics were calculated according to Bersier et al.^33^. For each taxon (*k*) within the different trophic levels, the diversity of individuals at lower trophic levels, i.e., primary parasitoids (*H*_*N*_, host diversity), and higher trophic levels, i.e., hyperparasitoids (*H*_*P*_, consumer diversity), were calculated as follows:

2 $${H}_{N,k}=-\mathop{\sum }_{i=1}^{S}\frac{{b}_{ik}}{{b}_{{\bullet }k}}\log _{2}\left(\frac{{b}_{ik}}{{b}_{{\bullet }k}}\right)$$

3 $${H}_{P,k}=-\mathop{\sum }_{i=1}^{S}\frac{{b}_{{kj}}}{{b}_{k{{\bullet }}}}\log_{2}\left(\frac{{b}_{ki}}{{b}_{k{\bullet}}}\right)$$

where *b*_*ik*_ is the number of individuals of primary parasitoid species *i* attacked by hyperparasitoid *k*, and *b.*_*k*_ is the total number of primary parasitoids (column sum of the parasitoid/host matrix) attacked by hyperparasitoid *k*; additionally, *b*_*kj*_ is the number of individuals of hyperparasitoid *j* attacking primary parasitoid *k*, and *b*_*k*_*.* is the total number of hyperparasitoids (row sum of the parasitoid/host matrix) attacking primary parasitoid *k*. The reciprocals of *H*_*N,k*_ and *H*_*P,k*_ are as follows:

4 $${n}_{N,k}=\left\{\begin{array}{cc}{2}^{{H}_{N,k}} & {{{{{\rm{if}}}}}}{b}_{{{\bullet}}k}\, > \,0\\ 0 & {{{{{\rm{if}}}}}}{b}_{{{\bullet}}k}=0\end{array}\right.$$

5 $${n}_{P,k}=\left\{\begin{array}{cc}{2}^{{H}_{P,k}} & {{{{{\rm{if}}}}}}{b}_{k{{\bullet}}}\, > \,0\\ 0 & {{{{{\rm{if}}}}}}{b}_{k{{\bullet}}}=0\end{array}\right.$$

G_q_ is the mean number of host species per consumer. A high G_q_ signals an increased number of host items for a given consumer, and Gq is calculated as follows:

6 $${{{{{{\rm{G}}}}}}}_{{{{{{\rm{q}}}}}}}=\left(\mathop{\sum }\limits_{k=1}^{S}\frac{{b}_{{{\bullet}}k}}{{b}_{{{\bullet}}{{\bullet}}}}{n}_{N,k}\right)$$

V_q_ is the mean number of parasitoid species per host. A high V_q_ signals that one host is parasitized by multiple species of consumers, and Vq is calculated as follows:

7 $${{{{{{\rm{V}}}}}}}_{{{{{{\rm{q}}}}}}}=\left(\mathop{\sum }\limits_{k=1}^{S}\frac{{b}_{k{{\bullet}}}}{{b}_{{{\bullet}}{{\bullet}}}}{n}_{p,k}\right)$$

C_q_ is the proportion of actual links of all possible links within the food web. Cq is quantified as follows:

8 $${{{{{{\rm{C}}}}}}}_{{{{{{\rm{q}}}}}}}=\frac{1}{2}\left(\mathop{\sum }\limits_{k=1}^{S}\frac{{b}_{k{{\bullet}}}}{{b}_{{{\bullet}}{{\bullet}}}}{n}_{P,k}+\mathop{\sum }\limits_{k=1}^{S}\frac{{b}_{{{\bullet}}k}}{{b}_{{{\bullet}}{{\bullet}}}}{n}_{N,k}\right)/s$$

where *s* is the number of species acting in the food web. A high C_q_ signals an increased availability of alternative resources for consumer populations.

Food web interactions were visualized using the “bipartite” package^74^ in R 4.0.2 software^73^. All variables are described in Supplementary Table 1.

### Statistical analysis

#### Landscape variables selection

First, we tested the correlations among five land-cover variables, i.e., cotton, maize, SC, NCH and urban areas, and landscape complexity (i.e., landscape diversity index, SIDI). The SIDI was highly correlated with maize cover (Supplementary Fig. 2a). To reduce the multicollinearity and simplify subsequent analyses, we performed PCA after excluding the SIDI and the urban land-cover category. The first two principal components (PCs) explained a total of 76% (43.8% of Dim1 and 32.1% of Dim2) of the variation in all dimensions. The first axis (PC1) mainly represented the land-cover category of maize, whereas the secondary axis (PC2) largely represented the NCH (Supplementary Fig. 2b). Pearson’s correlation test was conducted using the “ggcor” package^75^, and PCA was performed by the “vegan” package^76^ and visualized by the “factoextra” package^77^ of R 4.0.2 software^73^.

#### Direct effect analysis

To account for the direct effect of different predictors on response variables, we first performed multivariate regression analysis by using GLMs. In GLMs, we individually assessed predictors belonging to the same group, i.e., landscape composition (land-cover categories cotton, maize, SC, NCH), food web structural features (i.e., Gq, Vq, Cq), and diversity metrics (i.e., parasitoid or hyperparasitoid richness, diversity) on the ultimate ES (parasitism rate) or EDS (hyperparasitism rate). We selected the best-fit model based on the Akaike information criterion (AIC)^78^. Candidate models were selected with corrected AIC (*Δ*AICc < 4) due to small samples^79^, and the Akaike weight (*w*_i_) indicated the explanatory power of each model. The best-fit model was the one with the lowest AICc (*Δ*AICc = 0). The variance-inflation factor (VIF) values showed no significant multicollinearity (VIF < 2) between predictors in each group for the corresponding models.

Ecosystem functionality is usually not determined by a single factor; rather, it is typically determined by the combined effect of different factors^1,80^. Hence, LMMs were used to assess the direct effects of predictors from different functional groups (i.e., landscape factors, food web features, diversity of (hyper)parasitoid community). For parasitism, based on the previous direct analysis for the same functional group, fixed effects were included for three landscape predictors (cotton, SC, and NCH), one food web metric (Gq), species richness, and diversity of primary parasitoids (Supplementary Table 7). For hyperparasitism, fixed effects included three landscape variables (SC, NCH, and maize), hyperparasitoid richness and diversity, and three food web metrics (Cq, Gq, Vq) (Supplementary Table 9). “Year” was set as the random effect for model convergence. To select and infer the best model, we also performed conditional model averaging (*Δ*AICc = 0) from all candidate models (*Δ*AICc < 4), and we calculated the relative variable importance (importance) based on the summarized Akaike weight for each model (Supplementary Tables 8 and 10).

The VIF values were calculated within the “car” package;^81^ GLM analysis, LMM analysis and model selection and averaging were performed by using the “stats” package^73^, “lme4” package^82^ and “MuMIn” package^83^ of R 4.0.2 software^73^, respectively.

#### Cascaded effects assessment

Although regression analyses (GLMs or LMMs) showed direct effects of explanatory factors on response variables, they failed to clarify cascading relationships between these different variables. ES or EDS delivery can be simultaneously affected by land-use cover, species diversity of ES- or EDS-providing organisms and certain food web features^45,84,85^. To gauge how these different variables jointly mediated ecosystem functionality, a path analysis through piecewise SEMs was deployed^86^. Before the SEM path analysis, we tested the linear relationship between the parasitism rate (ES) and hyperparasitism rate (EDS). As no statistically significant patterns were recorded (Supplementary Fig. 3), we performed path analysis for the ultimate response variables, parasitism and hyperparasitism, individually.

Both SEM analyses were executed in two steps. For the parasitism rate (ES), we first entered all paths into the model and included the nonsignificant effect of landscape composition (i.e., NCH). Second, we filtered the nonsignificant landscape effect and retained only parasitoid diversity, food web Gq and species richness as predictors. Ultimately, path diagrams (Fig. 2a) were drawn to visualize the causal effects for different predictors on the target ES (i.e., parasitism rate). For the hyperparasitism rate (EDS), the primary model included two landscape variables (SC and NCH), hyperparasitoid richness and diversity, and food web Vq. Second, we removed nonsignificant effects on ecosystem functionality and reran the SEM analysis (Fig. 3a). Based on the directed separation tests, we tested a global goodness-of-fit with Fisher’s *C* statistic to determine model fitness and obtain the final model^87^ (Supplementary Tables 11 and 12). Finally, we calculated the cascading and total effect of each predictor, thus explaining its respective cascaded effects on ecosystem functionality (i.e., parasitism and hyperparasitism) based on the final models (Figs. 2a, 3a; Table 2). SEM analyses were performed by the “piecewise SEM” package^86^ of R 4.0.2 software^73^.

### Statistics and reproducibility

Statistical analysis of data was performed using R software as described above. For all statistical analysis, data from 25 independent measurements was used. The exact number of replicates are indicated in individual figure captions and the methods.

### Reporting summary

Further information on research design is available in the Nature Research Reporting Summary linked to this article.

## Supplementary information

Supplementary Information

Reporting summary

## Data Availability

All data generated in this study can be accessed from the Dryad Digital Repository. 10.5061/dryad.pc866t1kz^88^.
