# Supplementary material for: Species diversity and food web structure jointly shape natural biological control in agricultural landscapes
Source: Commun Biol. 2021 Aug 18;4:979. doi: 10.1038/s42003-021-02509-z (PMC8373963; doi:10.1038/s42003-021-02509-z)
Supplement: Supplementary file 1 — Supplementary Information [file 42003_2021_2509_MOESM1_ESM.pdf]

Supplementary information

Species diversity and food web structure jointly shape natural biological control in agricultural landscapes

Fan Yang[1,¶], Bing Liu[1,¶], Yulin Zhu[1,¶], Kris A.G. Wyckhuys[1], Wopke van der Werf[2], Yanhui Lu[1]*

[1] State Key Laboratory for Biology of Plant Diseases and Insect Pests, Institute of Plant Protection, Chinese Academy of Agricultural Sciences, Beijing, 100193, China.

[2] Centre for Crop Systems Analysis, Wageningen University and Research, 6708 PB Wageningen, The Netherlands.

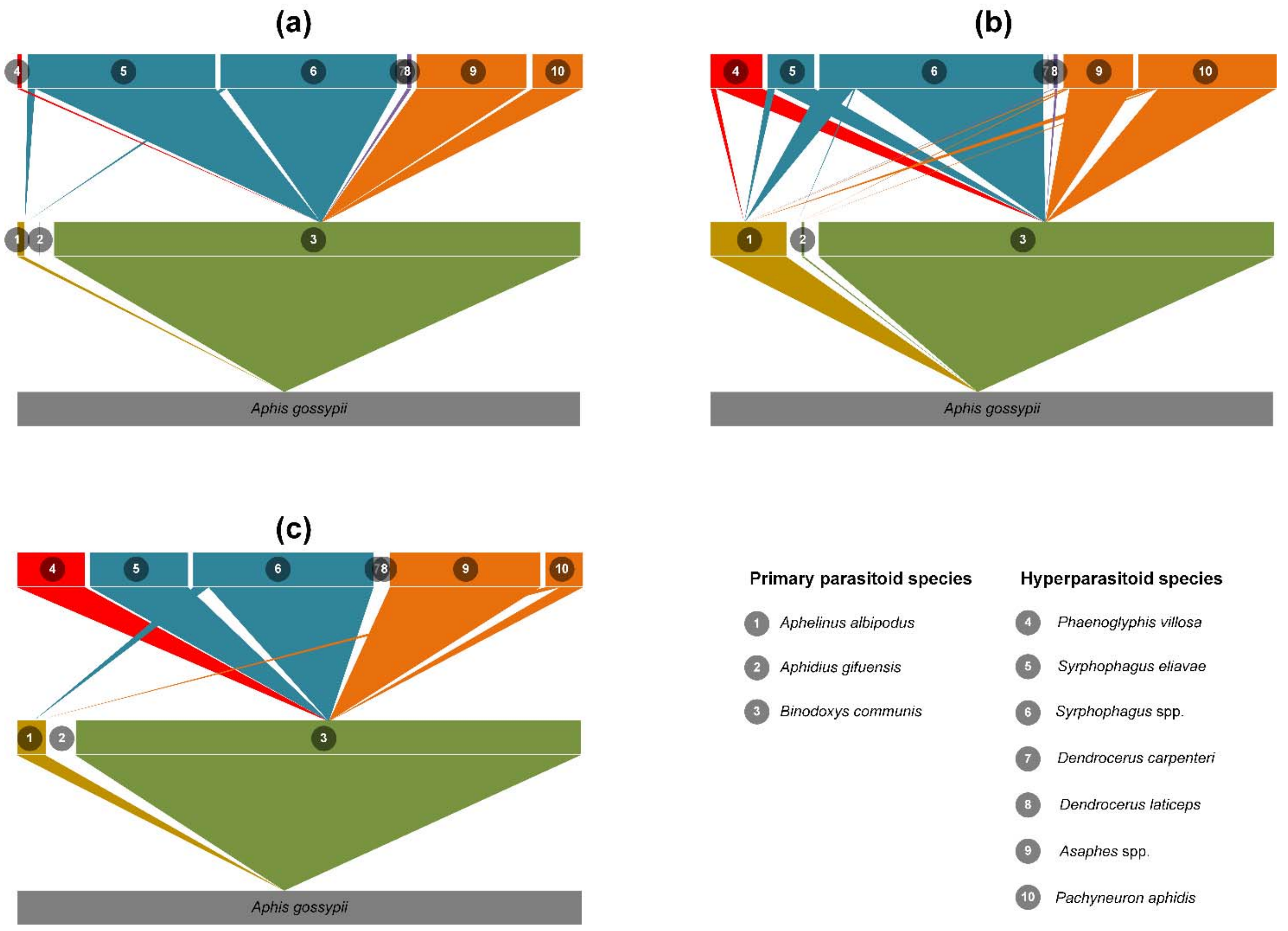

Supplementary Fig. 1. Quantitative host-primary parasitoid-hyperparasitoid food webs in different years. *Panels* **a**, **b**, and **c** show the tri-trophic food web in 2014, 2015 and 2016, respectively. In each panel, the bottom level represents the host (*A. gossypii*), the middle level indicates the primary parasitoids (species numbered circled with 1-3), and the highest level indicates the hyperparasitoids (species numbered circled with 4-10). Species that are marked with the same color belong to the same family. The width of a given triangle reflects the relative proportion of linkage effects.

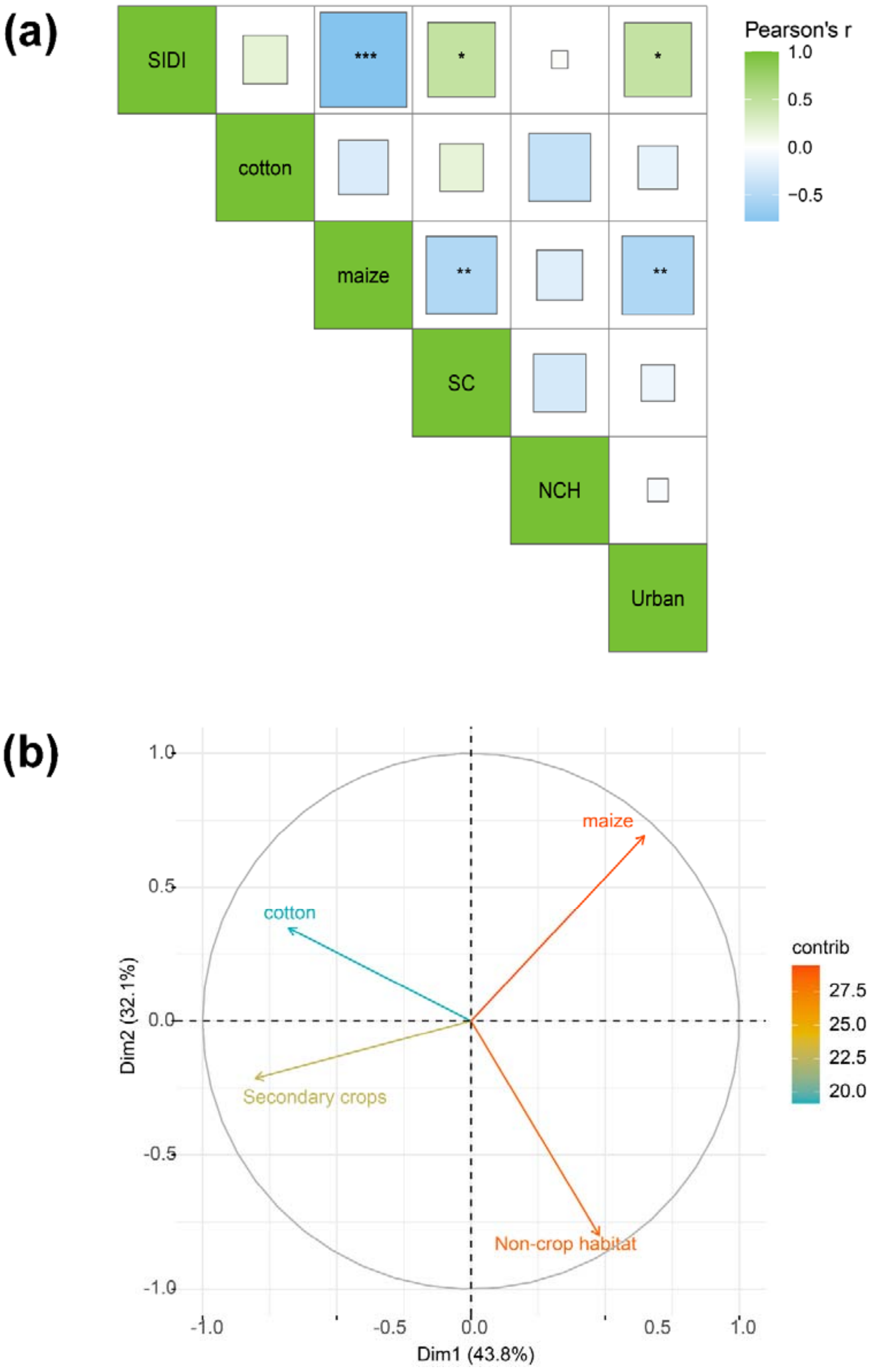

Supplementary Fig. 2. Landscape variable selection based on correlation and PCA. *Panel* **a** shows the correlation test of the original six landscape variables. *Panel* **b** shows the PCA of the four

landscape variables that were selected based on the correlation test. The color gradients of the landscape variables indicate their contributions to the PC axes (the first two principal components explained 43.8% and 34.1% of the variation, respectively). The landscape variables are detailed in the Methods and Supplementary Table 1.

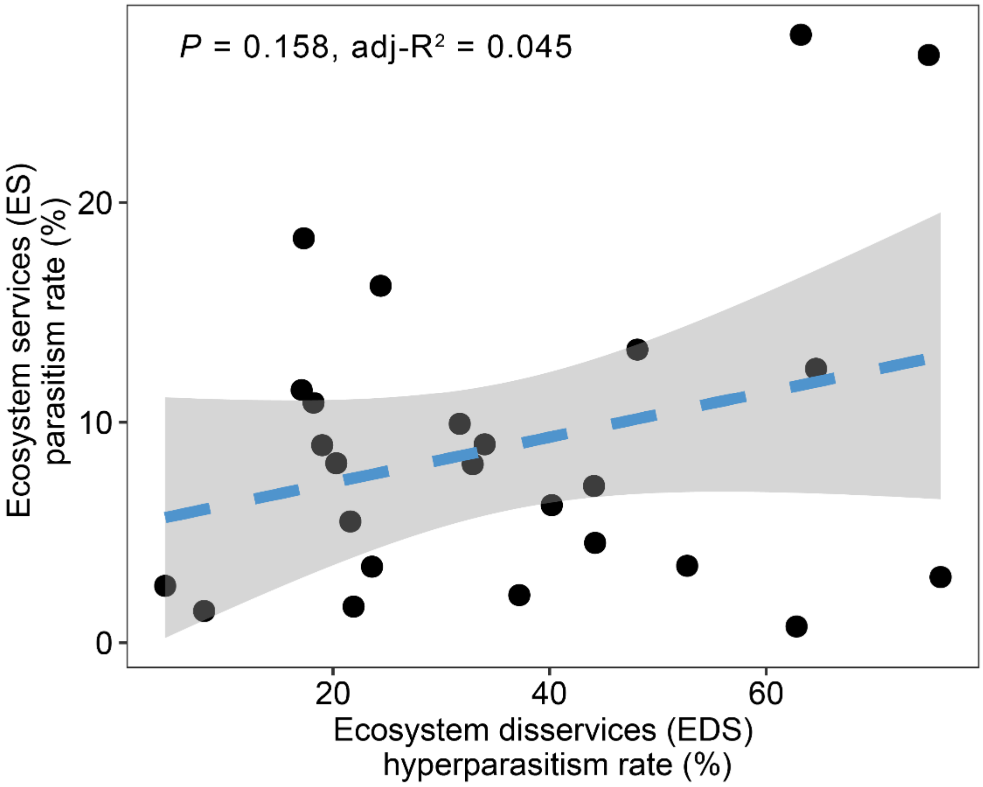

Supplementary Fig. 3. The linear relationship between the studied ES (parasitism rate) and EDS (hyperparasitism rate). Linear regression analysis showed that there was no significant linear relationship between the parasitism rate and hyperparasitism rate.

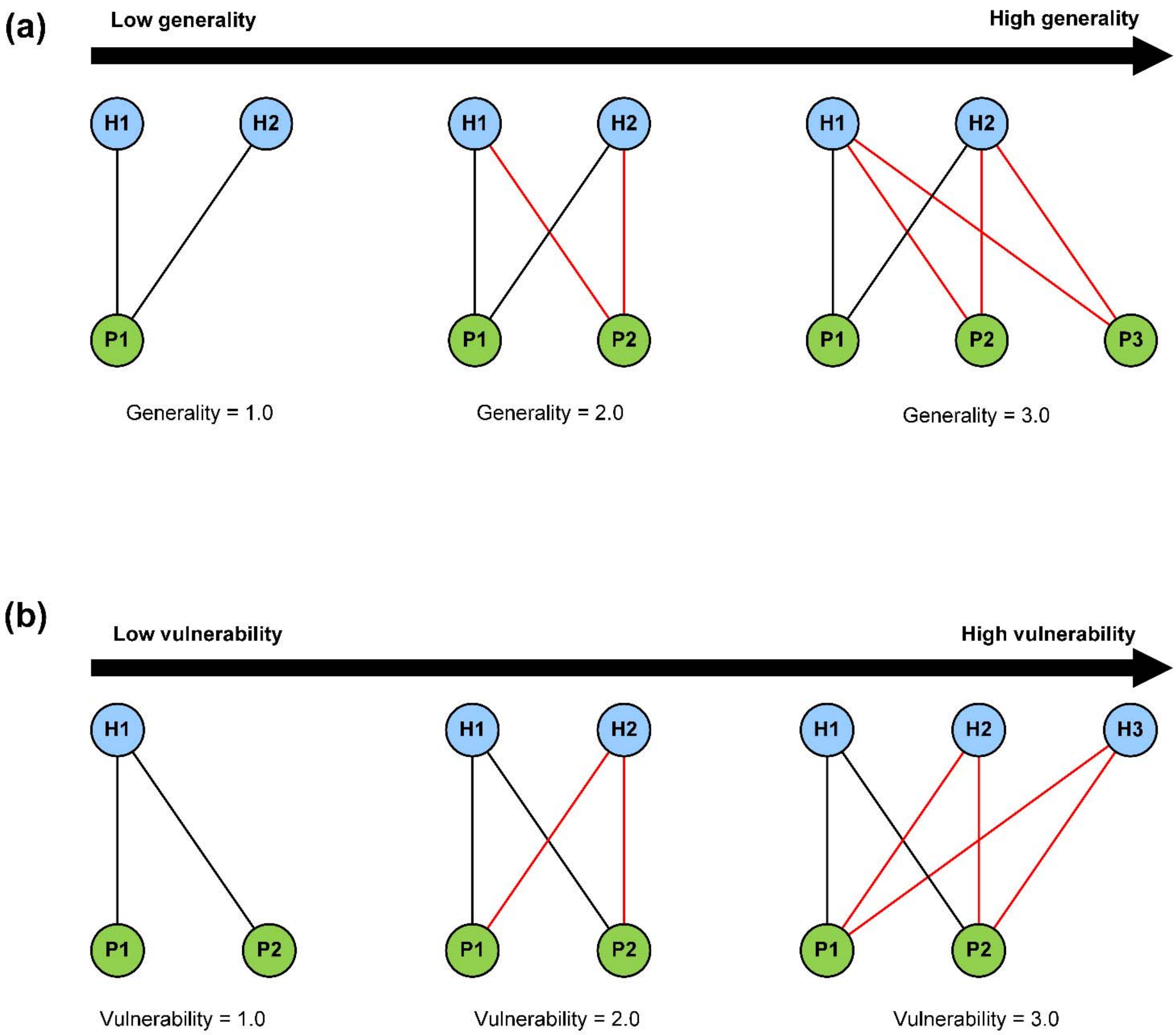

Supplementary Fig. 4. Conceptual model used to diagram the species interaction in the primary parasitoid-hyperparasitoid food web. *Panel* **a** shows that food web generality (Gq) increased with the species richness of primary parasitoids (hosts, at low levels) for each hyperparasitoid species (upper level). *Panel* **b** predicts a high food web vulnerability (Vq) because multiple species share one host species (primary parasitoid). Red links model the increased interactions between the upper level and lower level. Species numbered P1-P3 in the lower level (green circle) indicate primary parasitoids, whereas species H1-H3 (blue circle) represent hyperparasitoids.

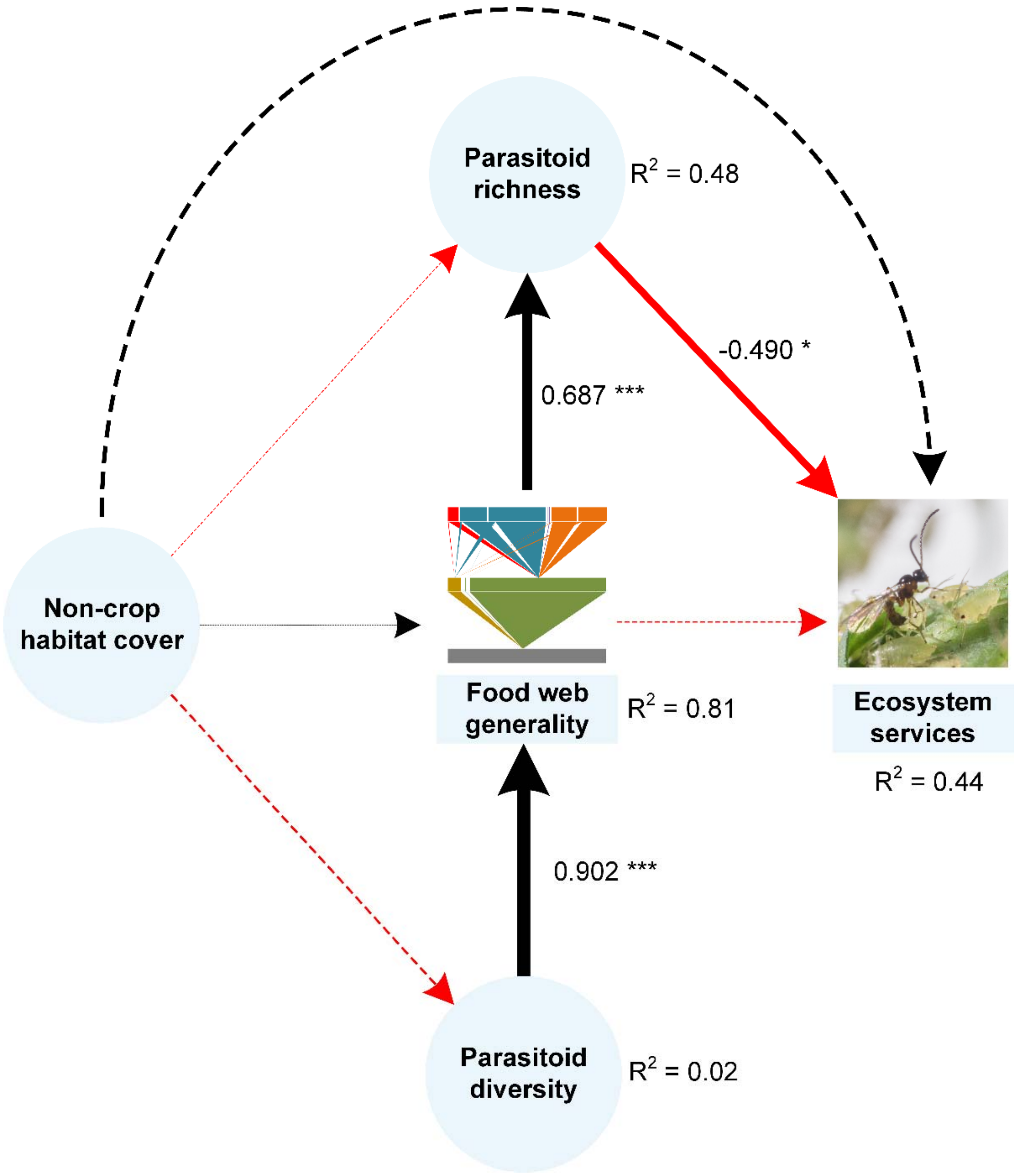

Supplementary Fig. 5. Causal paths between the ecosystem service (ES) of biological control and different (landscape, on-farm) predictors. In the SEM analysis, ES is the ultimate response variable, while parasitoid richness, parasitoid diversity and food web generality (Gq) are both predictors and response variables. Non-crop habitat cover (NCH) was the exogenous variable. The paths reveal both direct and indirect relationships between individual predictors and response variables. This diagram shows the initial path models with all variables. Standardized coefficients are shown for each path and scaled as line width. Black and red lines indicate either positive or negative relationships, with solid lines representing statistically significant effects and dotted lines showing

nonsignificant effects (* $P < 0.05$; ** $P < 0.01$; *** $P < 0.001$). R^2 shows the explanatory proportion of the total variance for each response variable in the model (Supplementary Table 11).

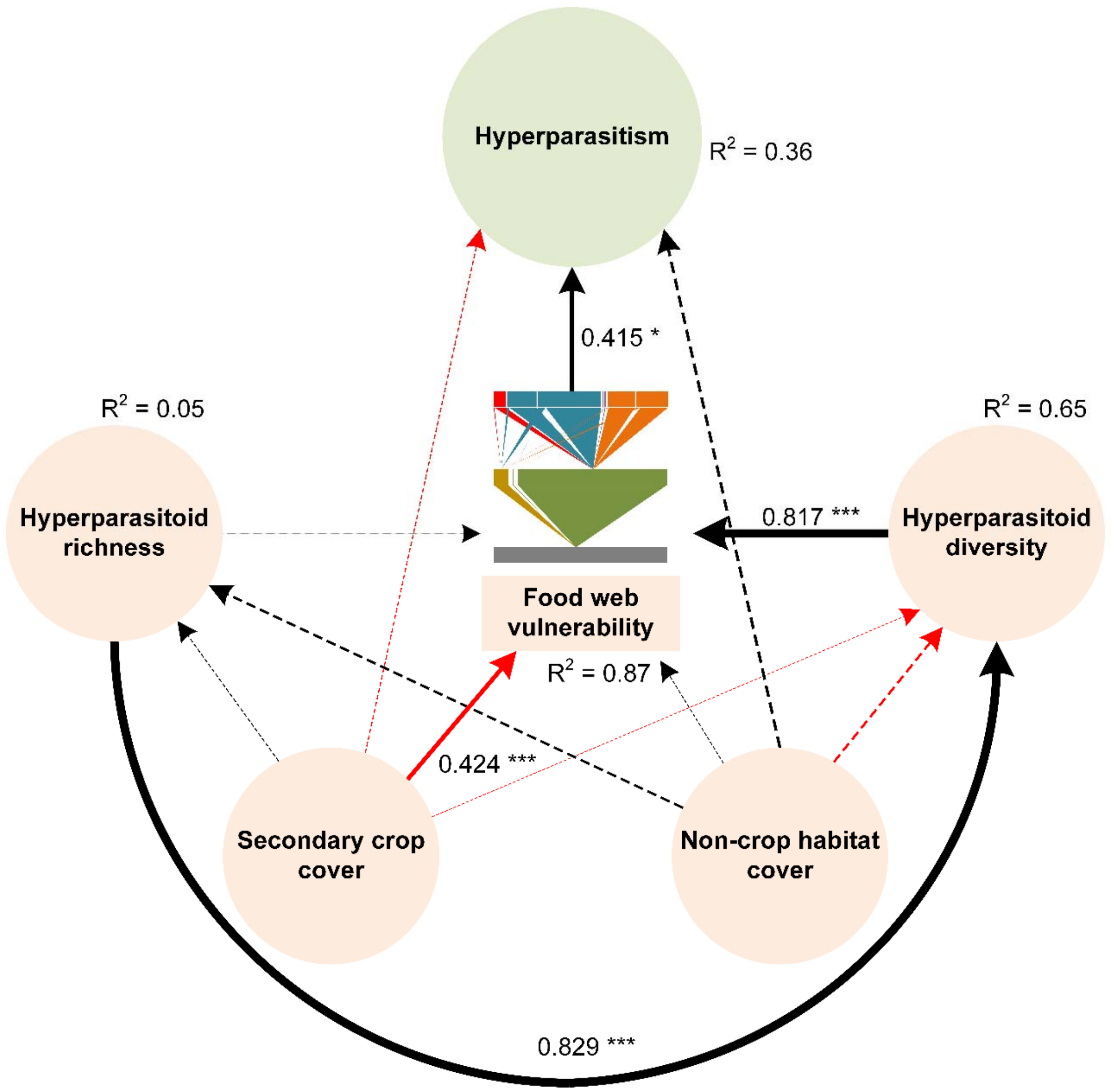

Supplementary Fig. 6. Causal paths between the ecosystem disservice (EDS) of hyperparasitism and different (landscape, on-farm) predictors. In the SEM analysis, EDS is the ultimate response variable, while hyperparasitoid richness and diversity and food web vulnerability (Vq) are both predictors and response variables. Non-crop habitat (NCH) and secondary crop cover (SC) are exogenous variables. The paths reveal both direct and indirect cascading relationships between predictors and response variables. This diagram shows the initial path models with all variables. Standardized coefficients are shown for each path and scaled as line width. Black and red lines indicate either positive or negative relationships, with solid lines representing significant effects and

dotted lines showing nonsignificant effects (* $P < 0.05$; ** $P < 0.01$; *** $P < 0.001$). R^2 shows the explanatory proportion of the total variance for each response variable in the model (Supplementary Table 12).

Supplementary Table 1. Variable descriptions for the study. Six factors indicated landscape composition and heterogeneity, four variables indicated the community metrics of (hyper)parasitoids (species richness and Shannon diversity), three metrics quantified the primary hyperparasitoid food web structure (Cq, Gq, Vq), and two indicators evaluated ecosystem functionality (ES and EDS).

Variable type	Variable name	Abbr.	Descriptions
Landscape factors	Simpson's inverse diversity index	SIDI	The index used to quantify the landscape heterogeneity.
	Proportion of cotton area	Cotton	The focal crop in the study region.
	Proportion of maize area	Maize	Another crop widely cultivated in northern China.
	Proportion of secondary crop area	SC	Secondary crops include peanut, soybean, sweet potato, vegetables and fruit trees.
	Proportion of non-crop habitat area	NCH	Non-crop habitats include grassland, shrubs and forest.
	Proportion of urban area	Urban	The combination of roads, cemented hard surfaces including buildings, water and abandoned land.
Parasitoid community metrics	Species richness	Parasitoid richness	The species richness of primary parasitoids from DNA detection.
		Hyperparasitoid richness	The species richness of hyperparasitoids from DNA detection.
	Shannon-diversity	Parasitoid diversity	Shannon-Wiener index (H´) that indicates the community diversity of primary parasitoids from DNA detection.
		Hyperparasitoid diversity	Shannon-Wiener index (H´) that indicates the community diversity of hyperparasitoids from DNA detection.
Food web features	Connectance	Cq	The weight proportion of actual links of all possible links within the food web.
	Generality	Gq	The weight mean number of host species per (hyper)parasitoid.

	Vulnerability	Vq	The weight mean number of (hyper)parasitoid species per host.
Ecosystem functionality	Ecosystem services (ESs)	Parasitism rate	The proportion of mummified aphids to all aphids in the field.
	Ecosystem disservices (EDSs)	Hyperparasitism rate	The proportion of hyperparasitoid detected from mummified samples.

Supplementary Table 2. Model selection for the effects of primary hyperparasitoid food web features on the studied ecosystem service (ES, parasitism rate) and disservice (EDS, hyperparasitism rate). The model selection and average were based on GLM analysis and ranked with the corrected Akaike information criterion (AICc). Delta is the difference in AICc ($\Delta < 4$ was marked as a candidate model), and the weight indicates the explanatory power of this model for the total variance. The top model has the smallest AICc and the highest weight. There are three quantitative metrics (Gq, Vq, Cq) used to quantize the architectonic features of the primary hyperparasitoid food web. The results showed that only Gq (generality of the food web) significantly affected the parasitism rate.

Response variable	(Intercept)	Cq	Gq	Vq	df	logLik	AICc	delta	weight
Parasitism rate	0.25		-0.13		3	33.74	-60.33	0.00	0.497
	0.28		-0.13	-0.01	4	34.02	-58.04	2.29	0.158
	0.28	-0.06	-0.14		4	33.76	-57.53	2.81	0.122
	0.09				2	30.74	-56.93	3.40	0.091
Hyperparasitism rate	0.02			0.12	3	8.54	-9.94	0.00	0.454
	0.29	-0.71		0.12	4	9.46	-8.92	1.02	0.272
	0.01		0.01	0.12	4	8.54	-7.08	2.85	0.109
	0.65	-1.09	-0.16	0.12	5	9.96	-6.75	3.18	0.092

Supplementary Table 3. Model selection for the effects of landscape composition on ecosystem functionality (parasitism rate, hyperparasitism rate). The model selection and average were based on GLM analysis and ranked with the corrected Akaike information criterion (AICc). Delta was the difference in AICc (Δ < 4 was marked as a candidate model), and the weight indicated the interpretability of this model for the total variance. The top model had the smallest AICc and the highest weight. Four landscape variables predicted the landscape composition quantized as the percentage of different land cover categories: cotton, maize, secondary crops (SC), and non-crop habitat (NCH). The results showed that only NCH only marginally significantly affected the parasitism rate and hyperparasitism rate.

Response variable	(Intercept)	cotton	maize	NCH	SC	df	logLik	AICc	delta	weight
Parasitism rate	0.05			0.25		3	32.62	-58.09	0.00	0.243
	0.09					2	30.74	-56.93	1.16	0.136
	0.03	0.22		0.32		4	33.40	-56.80	1.29	0.128
	0.09		-0.08	0.22		4	33.14	-56.28	1.81	0.099
	0.14		-0.11			3	31.64	-56.15	1.95	0.092
	0.04			0.27	0.07	4	32.77	-55.55	2.54	0.068
	0.08	0.05				3	30.79	-54.43	3.66	0.039
	0.09				0.00	3	30.74	-54.33	3.76	0.037
Hyperparasitism rate	0.05		0.44	0.88		4	8.77	-7.54	0.00	0.206
	0.26			0.73		3	6.73	-6.32	1.22	0.112
	0.43				-0.65	3	6.56	-5.98	1.56	0.094

0.33			0.58	-0.50	4	7.87	-5.73	1.81	0.083
0.48	-0.71			-0.55	4	7.79	-5.57	1.97	0.077
0.43	-0.87				3	6.36	-5.57	1.97	0.077
0.36					2	4.71	-4.88	2.66	0.054
0.33	-0.58		0.56		4	7.44	-4.87	2.67	0.054
0.10	-0.25	0.40	0.79		5	8.90	-4.64	2.90	0.048
0.10		0.38	0.82	-0.15	5	8.84	-4.52	3.02	0.045
0.23		0.32			3	5.63	-4.11	3.43	0.037
0.39	-0.52		0.43	-0.47	5	8.47	-3.77	3.77	0.031
0.33	-0.75	0.23			4	6.84	-3.67	3.87	0.030

Supplementary Table 4. Model selection for the effects of landscape composition on food web generality (Gq) and vulnerability (Vq). The model selection and averaging were based on GLM analysis and ranked with the corrected Akaike information criterion (AICc). Delta was the difference in AICc (*Δ* < 4 was marked as a candidate model), and the weight indicated the interpretability of this model for the total variance. The top model had the smallest AICc and the highest weight. Four landscape variables predicted the landscape composition quantized as the percentage of different land cover categories: cotton, maize, secondary crop (SC), and non-crop habitat (NCH). The results showed that only SC was significantly negatively related to Vq.

Response variable	(Intercept)	cotton	maize	NCH	SC	df	logLik	AICc	delta	weight
Gq	1.26					2	-1.30	7.15	0.00	0.185
	1.33	-0.94				3	-0.13	7.40	0.25	0.164
	1.28	-1.11			0.63	4	0.97	8.07	0.92	0.117
	1.46	-1.30		-0.69		4	0.85	8.29	1.14	0.104
	1.21				0.48	3	-0.73	8.60	1.45	0.090
	1.30			-0.29		3	-1.11	9.37	2.22	0.061
	1.26		-0.01			3	-1.30	9.75	2.60	0.050
	1.39	-1.37		-0.55	0.52	5	1.60	9.95	2.80	0.046
	1.39	-1.01	-0.13			4	-0.03	10.06	2.91	0.043
	1.64	-1.56	-0.33	-0.88		5	1.40	10.35	3.20	0.037
	1.10		0.22		0.64	4	-0.53	11.06	3.91	0.026
	1.22	-1.08	0.11		0.71	5	1.02	11.12	3.97	0.025
Vq	3.10				-3.21	3	-28.42	63.97	0.00	0.198

1.88		2.15			3	-28.55	64.24	0.27	0.173
1.43		2.46	2.27		4	-27.29	64.58	0.61	0.145
2.42		1.38		-2.17	4	-27.55	65.10	1.12	0.113
3.17	-1.09			-3.05	4	-28.25	66.50	2.53	0.056
2.76					2	-31.03	66.61	2.63	0.053
3.02			0.49	-3.07	4	-28.36	66.72	2.75	0.050
1.99	-0.86	2.04			4	-28.45	66.90	2.93	0.046
1.87		1.92	1.68	-1.31	5	-26.99	67.13	3.16	0.041
1.28	0.73	2.59	2.54		5	-27.23	67.62	3.65	0.032

Supplementary Table 5. Model selection for the effects of landscape composition on the species richness of primary parasitoids and hyperparasitoids. The model selection and averaging were based on GLM analysis and ranked with the corrected Akaike information criterion (AICc). Delta was the difference in AICc (*Δ* < 4 was marked as a candidate model), and the weight indicated the interpretability of this model for the total variance. The top model had the smallest AICc and the highest weight. Four landscape variables predicted the landscape composition quantized as the percentage of different land cover categories: cotton, maize, secondary crop (SC), and non-crop habitat (NCH). The results showed no landscape variable significantly affected the species richness of primary parasitoids and hyperparasitoids.

Response variable	(Intercept)	cotton	maize	NCH	SC	df	logLik	AICc	delta	weight
Parasitoid richness	1.84					2	-23.19	50.93	0.00	0.281
	1.71				1.25	3	-22.51	52.16	1.23	0.152
	1.92	-1.07				3	-22.94	53.02	2.09	0.099
	1.95			-0.80		3	-22.95	53.04	2.11	0.098
	1.82		0.04			3	-23.19	53.53	2.59	0.077
	1.80	-1.46			1.45	4	-22.02	54.04	3.11	0.059
	1.39		0.66		1.74	4	-22.20	54.39	3.46	0.050
	2.17	-1.75		-1.34		4	-22.35	54.71	3.78	0.043
	1.79			-0.46	1.12	4	-22.43	54.86	3.93	0.039
Hyperparasitoid richness	4.12					2	-36.27	77.08	0.00	0.288
	3.83			2.04		3	-35.71	78.56	1.48	0.138
	3.73		0.95			3	-35.98	79.10	2.02	0.105

	4.27	-1.89				3	-35.99	79.12	2.04	0.104
	4.09				0.27	3	-36.26	79.66	2.57	0.080
	3.24		1.29	2.48		4	-35.17	80.33	3.25	0.057

Supplementary Table 6. Model selection for the effects of landscape composition on the community diversity (Shannon diversity) of primary and hyperparasitoids. The model selection and average were based on GLM analysis and ranked with the corrected Akaike information criterion (AICc) (*Δ* < 4 was marked as candidate model), where delta was the difference in AICc, and weight indicated the interpretability of this model for the total variance. The top model had the smallest AICc and the highest weight. Four landscape variables predicted the landscape composition quantized as the percentage of different land cover categories: cotton, maize, secondary crop (SC), and non-crop habitat (NCH). The results showed that no landscape variable was significantly related to the Shannon diversity of the primary parasitoid or hyperparasitoid community.

Response variable	(Intercept)	cotton	maize	NCH	SC	df	logLik	AICc	delta	weight
Parasitoid diversity	0.22	-0.80			0.68	4	7.35	-4.70	0.00	0.163
	0.17				0.57	3	5.89	-4.63	0.07	0.158
	0.23					2	4.55	-4.55	0.16	0.151
	0.28	-0.61				3	5.33	-3.52	1.19	0.090
	0.38	-0.91		-0.57		4	6.39	-2.78	1.93	0.062
	0.30	-0.99		-0.42	0.59	5	7.96	-2.76	1.94	0.062
	0.27			-0.30		3	4.86	-2.57	2.14	0.056
	0.07		0.20		0.72	4	6.16	-2.32	2.38	0.050
	0.25		-0.06			3	4.57	-2.00	2.70	0.042
	0.19			-0.13	0.53	4	5.95	-1.91	2.80	0.040
	0.16	-0.76	0.12		0.76	5	7.46	-1.76	2.94	0.037
	0.55	-1.16	-0.31	-0.75		5	7.16	-1.15	3.55	0.028
	0.34	-0.69	-0.14			4	5.50	-1.00	3.71	0.026

Hyperparasitoid diversity	1.11					2	-1.90	8.34	0.00	0.304
	0.96		0.37			3	-1.19	9.52	1.18	0.169
	1.09	0.28				3	-1.81	10.75	2.41	0.091
	1.13			-0.09		3	-1.88	10.90	2.56	0.084
	1.11				0.04	3	-1.89	10.93	2.59	0.083
	0.85		0.53		0.44	4	-0.83	11.66	3.32	0.058
	0.90	0.50	0.43			4	-0.88	11.77	3.43	0.055

Supplementary Table 7. Candidate model selection for the effects of combined predictors on the studied ecosystem service (ES, parasitism rate). The candidate model selection and model average were based on LMM analysis and ranked with the corrected Akaike information criterion (AICc). Delta was the difference in AICc (*Δ* < 4 was marked as candidate model), and the weight indicated the interpretability of this model for the total variance. The top model had the smallest AICc and the highest weight. Predictors included landscape composition (NCH, cotton, SC), generality of the food web (Gq), species richness and the Shannon diversity of primary parasitoids.

(Intercept)	cotton	Gq	NCH	Richness	SC	Diversity	df	logLik	AICc	delta	weight
0.18			0.20	-0.06			5	37.89	-62.63	0.00	0.202
0.21				-0.07			4	36.10	-62.21	0.42	0.164
0.17			0.24	-0.07	0.14		6	38.99	-61.31	1.31	0.105
0.21				-0.07	0.08		5	36.45	-59.73	2.89	0.047
0.16	0.11		0.23	-0.06			6	38.18	-59.70	2.93	0.047
0.20		-0.02	0.20	-0.06			6	37.98	-59.29	3.34	0.038
0.24		-0.03		-0.06			5	36.21	-59.25	3.37	0.037
0.18			0.20	-0.06		-0.01	6	37.90	-59.13	3.50	0.035
0.21				-0.06		-0.02	5	36.14	-59.12	3.51	0.035
0.22	-0.02			-0.07			5	36.11	-59.07	3.56	0.034

Supplementary Table 8. Model-averaged coefficients and relative variable importance for the effects of combined predictors on the studied ecosystem service (ES, parasitism rate). The candidate model (ΔAICc < 4, in Supplementary Table 7) was conditionally averaged based on LMM analysis (sub-table **a**). The relative variable importance (importance) was calculated by summing the AICc for each model that included the predictor in all candidate models, where n represents the number of models that contained the predictor. For sub-table **b**, the top model had the minimum AICc ($\Delta = 0$), and the highest weight (20.2%) contained only two variables (species richness, NCH). The richness of primary parasitoids was significantly negatively related to the ES, whereas NCH was marginally positively related to the ES. Parasitoid richness = species richness of primary parasitoids; Gq = food web generality; Parasitoid diversity = community diversity of primary parasitoids; NCH = percentage of non-crop habitat cover, SC = percentage of secondary crop cover, cotton = percentage of focal crop cover.

(a): Model-averaged coefficients of conditional average and relative variable importance

Effects	Estimate	Std.Error	Adjusted.SE	z value	*P*	Importance	*n*
(Intercept)	0.192	0.047	0.049	3.93	**0.000**		
Parasitoid richness	-0.065	0.020	0.021	3.11	**0.002**	1.00	10
NCH	0.213	0.104	0.111	1.92	0.055	0.57	5
SC	0.126	0.101	0.108	1.17	0.242	0.20	2
cotton	0.056	0.157	0.166	0.34	0.736	0.11	2
Gq	-0.026	0.060	0.064	0.41	0.685	0.10	2
Parasitoid diversity	-0.013	0.072	0.077	0.17	0.864	0.09	2

(b): The fixed effects test of the top model ($\Delta = 0$)

Fixed effects	Estimate	Std.Error	df	t value	*P*
(Intercept)	0.178	0.039	25	4.59	**0.000**
Parasitoid richness	-0.064	0.018	25	-3.62	**0.001**
NCH	0.199	0.102	25	1.96	0.061

Supplementary Table 9. Candidate model selection for the effects of combined predictors on the studied ecosystem disservice (EDS, hyperparasitism rate). The candidate model selection and model average were based on LMM analysis and ranked with the corrected Akaike information criterion (AICc). Delta was the difference in AICc (*Δ* < 4 was marked as candidate model), and the weight indicated the interpretability of this model for the total variance. The top model had the smallest AICc and the highest weight. Predictors included landscape composition (NCH, cotton, maize, SC), food web metrics (Gq, Vq, Cq), species richness of hyperparasitoids (Richness_hyper), and the Shannon diversity of hyperparasitoids (Shannon_hyper). The top model showed that Vq was positively related to the hyperparasitism rate, whereas the NCH was marginally positively related to the hyperparasitism rate.

(Intercept)	Cq	Gq	maize	NCH	Richness _hyper	SC	Shannon _hyper	Vq	df	logLik	AICc	delta	weight
-0.02				0.57				0.11	5	10.15	-7.14	0.00	0.052
0.04								0.12	4	8.54	-7.09	0.05	0.051
0.07					0.09	-0.68			5	10.01	-6.86	0.28	0.045
0.36									3	6.90	-6.66	0.48	0.041
-0.06				0.68			0.29		5	9.65	-6.14	1.00	0.032
0.14							0.20		4	7.89	-5.79	1.35	0.026
0.29	-0.71							0.12	5	9.46	-5.76	1.38	0.026
0.29				0.46					4	7.80	-5.60	1.53	0.024
0.13					0.06				4	7.77	-5.54	1.60	0.023
-0.03				0.61		-0.51	0.32		6	11.07	-5.47	1.66	0.023
0.08						-0.67	0.31		5	9.26	-5.36	1.78	0.021

-0.21			0.34	0.87			0.27		6	11.00	-5.33	1.81	0.021
0.11						-0.32		0.10	5	9.00	-4.85	2.29	0.017
0.04				0.39	0.08	-0.57			6	10.75	-4.82	2.31	0.016
0.19	-0.55			0.51				0.11	6	10.74	-4.80	2.33	0.016
-0.03				0.58	0.07				5	8.95	-4.75	2.39	0.016
-0.05					0.04			0.09	5	8.90	-4.63	2.50	0.015
-0.07			0.23	0.68				0.09	6	10.65	-4.63	2.50	0.015
0.39						-0.32			4	7.25	-4.50	2.63	0.014
0.08			0.40	0.82					5	8.78	-4.40	2.74	0.013
-0.15			0.36	0.73	0.06				6	10.49	-4.31	2.82	0.013
0.49		-0.11							4	7.13	-4.26	2.88	0.012
-0.08				0.55	0.03			0.09	6	10.38	-4.09	3.05	0.011
0.31			0.11						4	7.02	-4.04	3.10	0.011
0.01			0.07					0.12	5	8.59	-4.02	3.12	0.011
0.03				0.53		-0.19		0.10	6	10.33	-3.99	3.15	0.011
0.04							-0.04	0.13	5	8.55	-3.95	3.19	0.011
0.06		-0.01						0.11	5	8.54	-3.93	3.21	0.010
0.38	-0.73					-0.70	0.30		6	10.28	-3.89	3.25	0.010
-0.07				0.62			0.10	0.08	6	10.25	-3.83	3.30	0.010
0.34	0.05								4	6.90	-3.81	3.33	0.010
-0.07		0.03		0.58				0.11	6	10.18	-3.70	3.44	0.009
0.04					0.07	-0.68	0.11		6	10.15	-3.64	3.50	0.009
0.04					0.07	-0.57		0.03	6	10.13	-3.59	3.54	0.009
0.42	-0.77					-0.37		0.10	6	10.12	-3.57	3.56	0.009
0.12		-0.05			0.09	-0.65			6	10.09	-3.51	3.63	0.008
0.07			0.00		0.09	-0.68			6	10.01	-3.35	3.79	0.008
0.07	-0.01				0.09	-0.68			6	10.01	-3.35	3.79	0.008
0.29		-0.13					0.21		5	8.23	-3.31	3.83	0.008

0.65	-1.09	-0.16		0.12	6	9.96	-3.25	3.89	0.007
0.28		-0.14	0.06		5	8.18	-3.21	3.93	0.007

Supplementary Table 10. Model-averaged coefficients and relative variable importance for the effects of combined predictors on the studied ecosystem disservice (EDS, hyperparasitism rate). The candidate model (ΔAICc < 4, in Supplementary Table 9) was conditionally averaged based on LMM analysis (sub-table **a**). The relative variable importance (importance) was calculated by summing the AICc for each model that included the predictor in all candidate models, where n represents the number of models that contained the predictor. For sub-table **b**, the top model that had the smallest AICc ($\Delta = 0$) and the highest weight contained only two variables (Vq, NCH). The Vq was significantly positively related to the response variable (EDS), whereas the NCH was marginally positively related to EDS. Vq, Gq, and Cq represent the quantitative food web metrics of vulnerability, generality and connectance, respectively; hyperparasitoid richness = species richness of hyperparasitoids; hyperparasitoid diversity = Shannon diversity of hyperparasitoid community; NCH = percentage of non-crop habitat cover, SC = percentage of secondary crop cover, maize = percentage of maize crop cover.

(a): Model-averaged coefficients of conditional average and relative variable importance

Effects	Estimate	Std. Error	Adjusted. SE	z value	*P*	Importance	n
(Intercept)	0.105	0.236	0.244	0.43	0.667		
Vq	0.107	0.049	0.052	2.05	**0.040**	0.41	17
NCH	0.611	0.338	0.357	1.71	0.087	0.40	15
SC	-0.555	0.341	0.359	1.55	0.122	0.29	14
Hyperparasitoid richness	0.070	0.041	0.043	1.63	0.103	0.27	13
Hyperparasitoid diversity	0.236	0.176	0.184	1.28	0.199	0.24	10
maize	0.246	0.255	0.267	0.92	0.358	0.13	7

Cq	-0.569	0.637	0.668	0.85	0.394	0.12	7
Gq	-0.076	0.162	0.171	0.45	0.655	0.09	7

(b): The fixed effects test of the top model (Δ = 0)

Fixed effects	Estimate	Std.Error	df	t value	*P*
(Intercept)	-0.02	0.11	25	-0.21	0.833
Vq	0.11	0.04	25	2.80	**0.010**
NCH	0.57	0.31	25	1.85	0.076

Supplementary Table 11. Path relationships between the studied ecosystem service (ES) and predictors based on SEMs. The ultimate response variable is the parasitism rate on aphids in cotton fields (ES). Endogenous variables are parasitoid diversity, richness, and generality (Gq) of the primary-hyperparasitoid food web, whereas the landscape factor (the percentage of non-crop habitat cover, NCH) is the exogenous variable. The Std. Estimate statistic is the standardized effect coefficient for each d-separated path model. The *P* value indicates the significance ($P < 0.05$) of each path. The last three rows with "~~" show the correlated error between three endogenous variables. The SEM analysis contained two steps: the top step contained the landscape variable (NCH), and the bottom step removed the landscape variable due to its nonsignificant effects on all response variables. The global goodness-of-fit of the whole model (Fisher's C = 0.67, df = 2, *P* = 0.716) and of the last model (Fisher's C = 0.65, df = 2, *P* = 0.723) were both fitted well and indicated they were reasonable models.

Response	Predictor	Estimate	Std.Error	DF	Crit.Value	*P*	Std. Estimate
			**Model contained landscape variable**				
Parasitism	richness	-0.06	0.03	21	-2.16	**0.043**	-0.490
Parasitism	Gq	-0.02	0.06	21	-0.38	0.708	-0.086
Parasitism	NCH	0.20	0.11	21	1.79	0.088	0.295
Richness	Gq	1.65	0.37	22	4.45	**0.000**	0.687
Richness	NCH	-0.32	0.89	22	-0.36	0.725	-0.055
Gq	diversity	1.14	0.12	22	9.55	**0.000**	0.902
Gq	NCH	0.05	0.23	22	0.20	0.843	0.019
Diversity	NCH	-0.30	0.39	23	-0.76	0.455	-0.156
~~Richness	~~Gq	0.69	-	25	4.45	**0.000**	0.688
~~Richness	~~diversity	0.03	-	25	0.14	0.444	0.030

~~Gq	~~diversity	0.90	-	25	9.55	**0.000**	0.898
**Removed landscape variable from the top model**							
Parasitism	richness	-0.06	0.03	21	-2.15	**0.043**	-0.524
Parasitism	diversity	0.03	0.14	21	0.18	0.859	0.072
Parasitism	Gq	-0.05	0.12	21	-0.38	0.705	-0.163
Richness	Gq	1.66	0.36	23	4.61	**0.000**	0.693
Gq	diversity	1.14	0.12	23	9.85	**0.000**	0.899

Supplementary Table 12. Path relationships between the studied ecosystem disservice (EDS) and predictors based on SEMs. The ultimate response variable is the hyperparasitism rate (EDS). Endogenous variables are hyperparasitoid richness, diversity, and vulnerability (Vq) of the primary-hyperparasitoid food web, whereas the percentage of non-crop habitat (NCH) and secondary crop (SC) are exogenous variables. The Std. Estimate statistic is the standardized effect coefficient for each d-separated path model. The *P* value indicates the significance ($P < 0.05$) of each path. The last three rows with "~~" show the correlated error between three endogenous variables (Vq, richness, diversity). The SEM analysis contained two steps: the top step contained multiple predictors, and the bottom step excluded all nonsignificant paths. The global goodness-of-fit of the top model (Fisher's C = 3.79, df = 4, *P* = 0.435) and the last model (Fisher's C = 6.21, df = 10, *P* = 0.797) were both fitted well and indicated the models were reasonable.

Response	Predictor	Estimate	Std.Error	DF	Crit.Value	*P*	Std. Estimate
			**Model contained multiple predictors**				
Hyperparasitism rate	Vq	0.10	0.05	21	2.14	**0.044**	0.415
Hyperparasitism rate	NCH	0.53	0.35	21	1.53	0.140	0.279
Hyperparasitism rate	SC	-0.19	0.35	21	-0.55	0.585	-0.110
Vq	richness	0.01	0.11	20	0.09	0.927	0.013
Vq	diversity	2.62	0.43	20	6.08	**0.000**	0.817
Vq	NCH	0.70	0.72	20	0.97	0.343	0.088
Vq	SC	-3.13	0.62	20	-5.07	**0.000**	-0.424
Diversity	richness	0.21	0.03	21	6.27	**0.000**	0.829
Diversity	NCH	-0.57	0.34	21	-1.68	0.107	-0.232

Diversity	SC	-0.17	0.31	21	-0.55	0.591	-0.074
Richness	NCH	2.32	2.11	22	1.10	0.285	0.237
Richness	SC	0.89	1.97	22	0.45	0.655	0.098
~~Diversity	~~Vq	0.00	-	25	0.00	0.500	0.000
~~Richness	~~Vq	0.00	-	25	0.00	0.500	0.000
~~Richness	~~diversity	0.00	-	25	0.00	0.500	0.000
**Removed nonsignificant paths from the top model**							
Hyperparasitism rate	Vq	0.12	0.04	23	2.87	**0.009**	0.514
Vq	diversity	2.64	0.25	22	10.59	**0.000**	0.824
Vq	SC	-3.31	0.57	22	-5.78	**0.000**	-0.449
Diversity	richness	0.20	0.03	23	5.94	**0.000**	0.778
