# Supplementary material for: Species diversity and food web structure jointly shape natural biological control in agricultural landscapes
Source: Commun Biol. 2021 Aug 18;4:979. doi: 10.1038/s42003-021-02509-z (PMC8373963; doi:10.1038/s42003-021-02509-z)
Supplement: Supplementary file 2 — Reporting summary [file 42003_2021_2509_MOESM2_ESM.pdf]

nature research

Corresponding author(s): Yanhui Lu

Last updated by author(s): Apr 1, 2021

# Reporting Summary

Nature Research wishes to improve the reproducibility of the work that we publish. This form provides structure for consistency and transparency in reporting. For further information on Nature Research policies, see our Editorial Policies and the Editorial Policy Checklist.

## Statistics

For all statistical analyses, confirm that the following items are present in the figure legend, table legend, main text, or Methods section.

| n/a | Confirmed | |
|---|---|---|
| ☐ | ☒ | The exact sample size (*n*) for each experimental group/condition, given as a discrete number and unit of measurement |
| ☐ | ☒ | A statement on whether measurements were taken from distinct samples or whether the same sample was measured repeatedly |
| ☐ | ☒ | The statistical test(s) used AND whether they are one- or two-sided<br>*Only common tests should be described solely by name; describe more complex techniques in the Methods section.* |
| ☐ | ☒ | A description of all covariates tested |
| ☐ | ☒ | A description of any assumptions or corrections, such as tests of normality and adjustment for multiple comparisons |
| ☐ | ☒ | A full description of the statistical parameters including central tendency (e.g. means) or other basic estimates (e.g. regression coefficient) AND variation (e.g. standard deviation) or associated estimates of uncertainty (e.g. confidence intervals) |
| ☐ | ☒ | For null hypothesis testing, the test statistic (e.g. *F*, *t*, *r*) with confidence intervals, effect sizes, degrees of freedom and *P* value noted<br>*Give P values as exact values whenever suitable.* |
| ☒ | ☐ | For Bayesian analysis, information on the choice of priors and Markov chain Monte Carlo settings |
| ☐ | ☒ | For hierarchical and complex designs, identification of the appropriate level for tests and full reporting of outcomes |
| ☐ | ☒ | Estimates of effect sizes (e.g. Cohen's *d*, Pearson's *r*), indicating how they were calculated |

*Our web collection on statistics for biologists contains articles on many of the points above.*

## Software and code

Policy information about availability of computer code

| | |
|---|---|
| Data collection | No software was used. |
| Data analysis | R version 4.0.2 for Windows (June-22-2020). |

For manuscripts utilizing custom algorithms or software that are central to the research but not yet described in published literature, software must be made available to editors and reviewers. We strongly encourage code deposition in a community repository (e.g. GitHub). See the Nature Research guidelines for submitting code & software for further information.

## Data

Policy information about availability of data

All manuscripts must include a data availability statement. This statement should provide the following information, where applicable:

- Accession codes, unique identifiers, or web links for publicly available datasets
- A list of figures that have associated raw data
- A description of any restrictions on data availability

All data is available on Dryad doi:10.5061/dryad.pc866t1kz

# Field-specific reporting

# Ecological, evolutionary & environmental sciences study design

All studies must disclose on these points even when the disclosure is negative.

| | |
|---|---|
| Study description | The study contained a 3-year study of quantitative parasitoid-hyperparasitoid trophic networks from 25 different agro-landscapes along with a gradient of crop and non-crop habitats. We assess the cascading effects of landscape composition, species diversity and trophic network structure on ecosystem functionality (i.e., parasitism, hyperparasitism).<br><br>General linear model (GLM) analysis with multiple model selection inference were first performed to assess the direct effects of explanatory variables belonging to the same groups (such as landscape composition, species diversity, quantitative food web metrics) on each response variable.<br><br>Linear mixed effect model (LMM) analysis then helped assess the direct effects of combinational predictors belonging to three groups: landscape composition, species richness and diversity, and food web features, on ESs and EDSs.<br><br>Path analysis with (structural equation models, SEMs) further reveals causality leading to the biological control of a resident crop pest, i.e., Aphis gossypii. Functionality is dictated by (hyper)parasitoid diversity, with its effects modulated by two key metrics of food web generality and vulnerability. Non-crop habitat cover directly benefits biological control, whereas secondary crop cover indirectly lowers hyperparasitism. |
| Research sample | A total of 25 cotton sites with different landscape heterogeneity were selected. For each study site, aphids numbers and the mummy aphids were sampled by direct observation, food web interactions of aphids-parasitoids were constructed and analysis basing on these mummy samples with DNA detection. |
| Sampling strategy | The 25 selected sites were further selected for independence between focal field and surrounding landscape context. Mummified (i.e., parasitized) aphids were collected over a 15-min sampling window in each plot (with 30 m transects) and individually placed within 1.5 mL centrifuge tubes with 95% ethanol for DNA detection to assess species diversity and interactions in aphid-primary parasitoid-hyperparasitoid food web. |
| Data collection | Over three years, we collected the data of landscape composition, we sampled mummy and recorded parasitism rate on aphid host in each focal field, the diversity of (hyper)parasitoids, the food web metrics (including three quantitative network metrics: generality, vulnerability, connectance), the hyperparasitism rate were derived from the mummy samples by using DNA detection and analysis.<br><br>A total of 2,153 mummified (i.e., parasitized) aphids were collected. DNA-based species identification and food web assembly revealed how 2,503 parasitoid and hyperparasitoid individuals (11 species) were involved in 2,386 distinct trophic interaction events. |
| Timing and spatial scale | These different sites across a landscape gradient in the > 3,000 km² cotton-growing region in Hebei and Tianjin provinces (northern China). From 2014 to 2016, 7-10 sites were selected each year and spaced at min. 3 000 m distance to avoid spatial autocorrelation. For each site, the landscape was digitized up to 1500 m radius scale from the focal cotton field. Sampling was carried out three times (seven days interval) in each field from early July to mid-August in each year when the parasitism was significant occurred. |
| Data exclusions | No data exclusions. |
| Reproducibility | Statistical analysis was fully reproduced upon analysis of all data. |
| Randomization | The 25 landscape study sites were selected randomly across the main production areas. Aphids and mummies were recorded on 50 cotton plants in each of three randomly-selected cotton plots (min. 1,000 m²) within the focal cotton field at each site. Within each plot, five points were randomly chosen using a Z-shaped sampling grid and 10 plants were inspected at each point. Sampling was carried out three times in each field from early July to mid-August. |
| Blinding | All experiments and analyses were performed double blind - each study site was assigned a unique identifier. |

Did the study involve field work? ☒ Yes ☐ No

## Field work, collection and transport

| | |
|---|---|
| Field conditions | The study was conducted from 2014 to 2016 in the main cotton-growing region in Hebei and Tianjin province in northern China plain. The study region covers an area of approximately 3000 km² and is mostly dominated by smallholding managed arable land (approx. 55%) and non-crop habitats (approx. 15%) and urban (approx. 30%). For this study, 25 sites were selected to cover a size gradient of landscape heterogeneity. All focal cotton fields (Bt cotton variety "guo xin", commonly cultivated in the study area) had similar agronomic management practices. Sampling was exclusively done in insecticide-free cotton plots. The focal fields were managed without pesticides during the whole study period and farmers were financially compensated for any yield loss that resulted from this modified pest management regime. All field assays having similar phenological and meteorological conditions. The mean temperature during sampling was 25°C. |
| Location | The study sites were located in the main cotton production area of northern China plain (116°28′ E- 116°56′ E, 38°46′N - 39°36′ N), near the Langfang city in Hebei Province and Wuqing, Jinghai, Ninghe cities in Tianjin Province from 2014 to 2016. The average altitude of the sampled areas was 20 m. |
| Access & import/export | All sampling sites were accessible by car using public roads. All field work was done with due authorization from the landowners. |

| | |
|---|---|
| Disturbance | The study sites and organsims were not disturbed by any other means than the sampling, which was restricted to 30 m wide transects in each 1000 $m^2$ plot. |

# Reporting for specific materials, systems and methods

We require information from authors about some types of materials, experimental systems and methods used in many studies. Here, indicate whether each material, system or method listed is relevant to your study. If you are not sure if a list item applies to your research, read the appropriate section before selecting a response.

## Materials & experimental systems

| n/a | Involved in the study | |
|---|---|---|
| x | | Antibodies |
| x | | Eukaryotic cell lines |
| x | | Palaeontology and archaeology |
| | x | Animals and other organisms |
| x | | Human research participants |
| x | | Clinical data |
| x | | Dual use research of concern |

## Methods

| n/a | Involved in the study | |
|---|---|---|
| x | | ChIP-seq |
| x | | Flow cytometry |
| x | | MRI-based neuroimaging |

# Animals and other organisms

Policy information about studies involving animals; ARRIVE guidelines recommended for reporting animal research

| | |
|---|---|
| Laboratory animals | NA |
| Wild animals | Cotton aphids were sampled with visual inspection and released in the field as described above. The mummy aphids were sampled in field and individually placed within 1.5 mL centrifuge tubes with 95% ethanol for the parasitoids diversity identification and aphids-parasitoids food web interaction detection. |
| Field-collected samples | For each study site, mummy aphids were collected and individually placed within 1.5 mL centrifuge tubes with 95% ethanol, and subsequently kept at -20˚C for subsequent PCR-based parasitoid identification. We identified aphids and parasitoids (including primary and hyperparasitoids) to species level. |
| Ethics oversight | No ethical oversight was required because the host aphids (Aphis gossypii) was a common pest on cotton, and was usually acted as a model pest in ecological study. |

Note that full information on the approval of the study protocol must also be provided in the manuscript.
